# Evolution of Indian Influenza A (H1N1) Hemagglutinin Strains: A Comparative Analysis of the Pandemic Californian HA Strain

**DOI:** 10.3389/fmolb.2023.1111869

**Published:** 2023-03-16

**Authors:** Shilpa Sri Pushan, Mahesh Samantaray, Muthukumaran Rajagopalan, Amutha Ramaswamy

**Affiliations:** ^1^ Department of Bioinformatics, Pondicherry University, Puducherry, India; ^2^ Department of Biological Sciences and Bioengineering, Indian Institute of Technology Kanpur, Kanpur, India

**Keywords:** influenza A, H1N1, virus, phylogenetic analysis, antigenic site, N-glycosylation, receptor-binding domain

## Abstract

The need for a vaccine/inhibitor design has become inevitable concerning the emerging epidemic and pandemic viral infections, and the recent outbreak of the influenza A (H1N1) virus is one such example. From 2009 to 2018, India faced severe fatalities due to the outbreak of the influenza A (H1N1) virus. In this study, the potential features of reported Indian H1N1 strains are analyzed in comparison with their evolutionarily closest pandemic strain, A/California/04/2009. The focus is laid on one of its surface proteins, hemagglutinin (HA), which imparts a significant role in attacking the host cell surface and its entry. The extensive analysis performed, in comparison with the A/California/04/2009 strain, revealed significant point mutations in all Indian strains reported from 2009 to 2018. Due to these mutations, all Indian strains disclosed altered features at the sequence and structural levels, which are further presumed to be associated with their functional diversity as well. The mutations observed with the 2018 HA sequence such as S91R, S181T, S200P, I312V, K319T, I419M, and E523D might improve the fitness of the virus in a new host and environment. The higher fitness and decreased sequence similarity of mutated strains may compromise therapeutic efficacy. In particular, the mutations observed commonly, such as serine-to-threonine, alanine-to-threonine, and lysine-to-glutamine at various regions, alter the physico-chemical features of receptor-binding domains, N-glycosylation, and epitope-binding sites when compared with the reference strain. Such mutations render diversity among all Indian strains, and the structural and functional characterization of these strains becomes inevitable. In this study, we observed that mutational drift results in the alteration of the receptor-binding domain, the generation of new variant N-glycosylation along with novel epitope-binding sites, and modifications at the structural level. Eventually, the pressing need to develop potentially distinct next-generation therapeutic inhibitors against the HA strains of the Indian influenza A (H1N1) virus is also highlighted here.

## 1 Introduction

Influenza is a global viral threat that can lead to severe or fatal diseases. It targets every class of individuals, including pregnant women and immunocompromised people (Cox and Subbarao, 2000; Rambaut et al., 2008; Makau et al., 2017). According to the World Health Organization (WHO), there have been approximately 3–5 million cases of influenza each year since 2009, with over 650,000 deaths (Iuliano et al., 2017; Jones et al., 2019). Commonly, the epidemic of influenza is highly reported in the winter season of the temperate zone. It not only affects individuals but also causes significant economic losses due to several factors including workplace absenteeism and costs of the treatment (Simonsen, 1999; Gatherer, 2009). The notable concern is the virulence of the influenza A viruses causing global pandemics. The pandemic outburst of influenza A (H1N1) in 2009 is the latest episode reported in the last decade (Garten et al., 2009; Intelli-et al., 2009; Mishra et al., 2010). In 2009, during the pandemic outbreak of the influenza A (H1N1) pdm09 strain, India reported about 27,236 virology laboratory-certified cases of influenza A (H1N1) with 981 fatal reports (https://www.ncdc.gov.in/dashboard.php). The WHO documented that the pandemic virus would continue as the seasonal influenza virus (WHO, 2010). The Ministry of Health and Family Welfare reported in 20 October 2020 that in the post-pandemic period (i.e., since 2010), the influenza A (H1N1) pdm09 strain caused nearly 185,578 laboratory-confirmed cases with more than 12,000 deaths in India. The maximum cases were reported from states like Rajasthan, Gujarat, Delhi, Jammu and Kashmir, Maharashtra, Madhya Pradesh, Telangana, Karnataka, and Tamil Nadu (Dashboard:: National Centre for Disease Control (NCDC). The periodic outbreak of influenza poses critical challenges in the public health.

In particular, the flu viruses, belonging to the Orthomyxoviridae family, are classified as influenza A, B, C, and D types. Influenza A-type viruses are reported to cause infection in multiple hosts, like avian and mammalian species, while the B-type influenza infection is restricted to humans (Paules and Subbarao, 2017; Ghaffari et al., 2019; Ravina et al., 2020). Influenza C causes a mild infection in humans but is not either epidemic or pandemic in nature. The type D-mediated flu is mainly reported in cattle and pigs but not in humans (Odagiri et al., 2015; Zhai et al., 2017). The genomes of influenza viruses A and B contain eight negative-sense single-stranded RNA (-ssRNA) segments, whereas those of influenza viruses C and D contain only seven -ssRNA segments due to the absence of one of the envelope glycoproteins (Wang and Veit, 2016; Su et al., 2017). The RNA segments of influenza A and B viruses with negative polarity encode about 10 proteins, namely, 1) two surface glycoproteins (hemagglutinin (HA) and neuraminidase (NA)), 2) one nucleoprotein (NP), 3) three polymerase proteins (PA, PB1, and PB2), 4) two matrix proteins (M1 and M2), and 5) two non-structural proteins (NS1 and NS2) (Dandagi and Byahatti, 2011; Murhekar and Mehendale, 2016; Lazniewski et al., 2018; Chua et al., 2019). The C and D influenza viral RNA segments code for nine proteins due to the lack of envelope glycoproteins (Ferguson et al., 2016; Asha and Kumar, 2019). Among these proteins, both HA and NA disclose 18 and 11 subtypes of surface proteins, respectively. These surface proteins play a crucial role in the naming of viral diseases (Webster and Govorkova, 2014; Chua et al., 2019).

HA is a central factor in the initialization of the infection and responsible for binding of the virus to the host cell receptor (sialic acid) surface. HA promotes the fusion of the virus membrane with the host endosomal membranes to facilitate viral entry into the host cell (Saxena et al., 2018). Another surface glycoprotein NA intercepts the newly synthesized virion concentration by breaking the alpha-ketosidic linkage between sialic acid and the proximate sugar residue in order to stop 1) virion aggregation and 2) the virus binding back to the dying host cell *via* HA. This allows for the efficient release of viral progeny, which then spreads to new target cells. This results in the disruption of the identification of the HA receptor-binding site and facilitates the spread of viral particles beyond the infected site and promotes severe infection (McAuley et al., 2019). Previous studies suggest the phenotypic variation is guided by a series of mutations that change the antigenic properties of the strain (McDonald et al., 2007; Sriwilaijaroen and Suzuki, 2012). The majority of the antigenic drift in the influenza virus is thought to be guided by the mutations in the HA1 region of the HA protein (Wiley et al., 1981; Nelson and Holmes, 2007).

The evolution of influenza strains is mainly driven by the antigenic drift due to frequent and continuous mutations. With such dynamic antigenic changes, the virus continuously and steadily multiplies and accumulates in the cell/organism (Lin et al., 2009; Neumann et al., 2009; Shi et al., 2010). Variations generated by the mutations mainly affect the affinity or specificity of both antigenic and receptor-binding sites (Gerhard et al., 1981; Yokoyama et al., 2017), and also mediate conformational changes in the receptor-binding pocket as well (Sriwilaijaroen and Suzuki, 2012). With all these possible mutations, the virus becomes insensitive to the inhibitors, which were designed specifically for the native strains. Viruses with such significant variability pose a severe challenge to society, especially in the diagnosis, medication, and control of viral infection in humans (Sriwilaijaroen and Suzuki, 2012; Hütter et al., 2013; Alonso et al., 2015; Guillebaud et al., 2017; Sharma et al., 2019). Hence, it is important to study the mutational and phylogenetic evolution of the HA surface protein from different strains of the influenza virus, especially by characterizing the recognition sites such as the receptor-binding site, N-glycosylation site, and the antigenic sites.

The current study implements comparative sequence analyses to characterize and establish the evolutionary relationships of Indian isolates with the pandemic Californian strain (being the closest member to these Indian strains) reported in 2009 as a reference to describe changes reported in the swine (H1N1) virus during 2009–2018. *In silico* analysis is performed by comparing the HA protein sequences of the Indian influenza A (H1N1) virus to the reference pandemic strain (A/California/04/2009) with special emphasis on the characterization of various recognition sites including receptor-binding sites, antigenic binding sites, and glycosylation sites, by accounting the variants reported since 2009.

Hereafter, the isolates of HA protein sequences of the influenza A (H1N1) virus infecting humans from California and India will be referred to as HA_Cal_ and HA_Ind_, respectively, throughout the article. For the structural analysis, two representative structures, namely, 1) HA_Cal_ (reported in 2009, the reference strain) and 2) the HA protein (Acc No: QCP70896) reported in 2018 (HA_Ind-2018_) have been used in this study.

## 2 Material and methods

### 2.1 Data collection

The HA_Ind_ protein sequences, reported from various geological locations of India during 2009–2018, are deposited in the NCBI (Table 1), and the same were retrieved for the present study (https://www.ncbi.nlm.nih.gov/genomes/FLU/Database/nph-select.cgi?go=database).

**TABLE 1:** NCBI accession numbers of the HA_Ind_ strains of the influenza A (H1N1) virus reported during 2009–2018.

**Year**	**2009**	**2010**	**2011**	**2012**	**2013**	**2014**	**2015**	**2016**	**2017**	**2018**
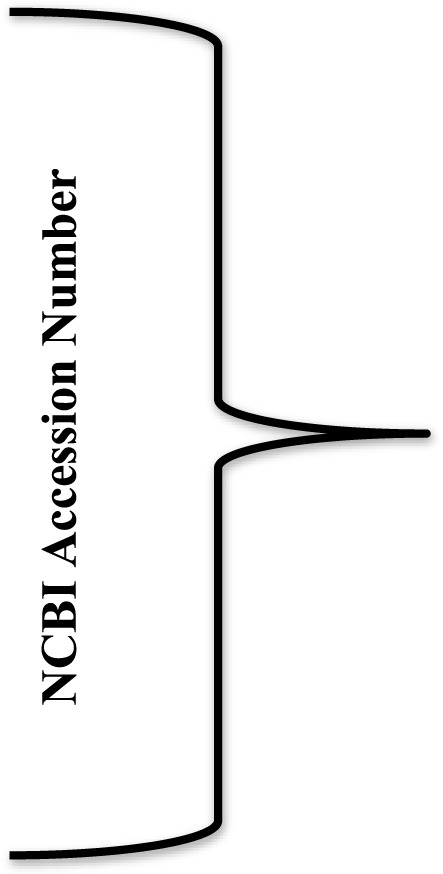	AEN79398	AJE62461	AKM14732	AKM14739	AJE62527	AKE37501	ALA50342	ASJ82233	ATW75053	QEU44874
	ALX27941	AEN79399	AKM14733	AKM14740	AJE62528	AKE37493	ALA50343	ASJ82234	ATW75054	QEU44876
	ALX27940	AEM63501	AKM14734	AKM14741	AJE62529	AKE37494	ALD18975	ASJ82238	ATW75055	QEU44877
	AKM14710	AKM14717	AKM14735	AKM14742	AJE62530	ARG42801	AKS48057	ASJ82239	ATW75056	QEU44883
	AKM14711	AKM14718	AKM14736	AKM14743	AJE62520	ARG42802	ALK80387	ASJ82240	ATW75057	QEU44880
	AKM14712	AKM14719	AIU46629	AKM14744	AJE62521		ALK80385	ASJ82241	ATW75058	QEU44882
	AKM14713	AKM14720	AKS48053	AKM14745	AJE62522		ALK80389	ASJ82242	ATW75059	QEU44879
	AKM14714	AKM14721	AJE62491	AKM14746	AKE37409		ALK80390	ASJ82243	ATW75060	QEU44878
	AKM14715	AKM14722	AJE62492	AKM14747	AGY42549		ALK80386	ASJ82244	ATW75061	QEU44873
	AKM14716	AKM14723	AJE62493	AKM14748	AKE37418		ALK80388	ASJ82245	ATW75062	QPC70893
	------------	------------	------------	------------	------------	------------	------------	------------	------------	------------
	------------	------------	------------	------------	------------	------------	------------	------------	------------	------------
	-------------	-------------	-------------	-------------	-------------	-------------	-------------	-------------	-------------	-------------
	AJE62445	AJE62474	AJE62483	AII31198	AJE62523	------------		ASU06458	ASR91934	QCP70895
	ADG57095	AEM63474	AJE62484	AJE62511	AJE62524	-------------		ASU06459	ASR91935	QCP70896
**Total no. of isolates**	**134**	**58**	**33**	**93**	**27**	**05**	**53**	**22**	**74**	**13**

### 2.2 HA sequence retrieval and multiple sequence analysis (MSA)

To understand the mutational and evolutionary drift among the HA_Ind_ protein sequences that circulated during the aforementioned period, MSA was carried out using ClustalW (Thompson et al., 1994). The evolutionarily closest sequence has been identified using the pair-wise distance matrix method. The resulting MSA was used to find the evolutionarily conserved regions in the examined sequences.

### 2.3 Evaluation of the phylogenetic relation

Following MSA, phylogenetic analysis was performed on HA_Ind_ proteins reported during 2009–2018. In order to understand the evolutionary relationship of these HA_Ind_ proteins of H1N1 strains from India, along with the reported pandemic Californian strain (A/California/04/2009), a phylogenetic tree was constructed using the maximum parsimony method. Parsimony analysis was performed in PAUP (v.4.0) using a heuristic search approach along with the following settings: 1) characters unordered with equal weight, 2) random taxon addition, and 3) branch swapping with the tree bisection–reconnection (TBR) algorithm. Resampling was performed with 1,000 replicates by bootstrapping to check the reliability of the results (Felsenstein, 1985; Tamura et al., 2004; Victoria Martínez et al., 2008). The selected HA_Ind_ sequences revealed a close relationship with the pandemic HA_Cal_ protein of the pandemic strain (A/California/04/2009) reported in 2009. Hence, the HA_Ind_ sequences, evolved as the closest members to the HA_Cal_ protein, were clustered as one clade and used for further studies (Table 2).

**TABLE 2:** Identified top 10 HA_Ind_ proteins share the closest evolutionary relationship with the reference HA_cal_ strain.

**List of Indian isolates closest to the Californian strain**			
**Year**	**Gene accession number**	**Variant’s name**	**Abbreviated variant’s name used in the study**
2009	GQ280797	A/California/04/2009	CA-HA-09
2009	AEM63482	A/Blore/NIV1196/2009	IN-HA-09
2010	AIU46627	A/Khargone/293/2010	IN-HA-10
2011	AEX63612	A/India/GWL01/2011	IN-HA-11
2012	AJE62498	A/Delhi/057/2012	IN-HA-12
2013	AKE37409	A/India/Alp135125/2013	IN-HA-13
2014	ARG42801	A/Kerala/RGCB140815/2014	IN-HA-14
2015	ALK80385	A/India/DRDE_GWL719/2015/	IN-HA-15
2016	ASJ82246	A/India/P167512/2016	IN-HA-16
2017	ASJ82235	A/India/P1722376/2017	IN-HA-17
2018	QCP70896	A/India/Che-1851811/2018	IN-HA-18

### 2.4 Mutational analysis

Mutational analysis was carried out using the ClustalW alignment tool of BioEdit (version 7.2.5) (Hall, 1999) with a bootstrap value of 1,000 to generate a global alignment for the selected HA_Ind_ proteins compared with the HA_Cal_ protein (Table 2) to investigate whether there is any prevalence of phenotypic variation in the reported HA_Ind_ protein sequences. The algorithm computed a distance matrix between each pair of sequences based on pairwise sequence alignment scores.

### 2.5 *Ab initio* structural modeling of the HA protein

In addition to sequence comparison, the effects of mutations on the structure of HA were also investigated. For the HA structure comparison analysis, the following were selected: 1) one of the Indian HA proteins (Acc No: QCP70896), reported in 2018 (will be referred to as HA_Ind-2018_, hereafter) and 2) the reference HA_Cal_ protein.

The complete crystallographic structure of the reference HA_Cal_ protein (with 566 amino acids) is not reported in the PDB website, and the reported HA_Cal_ structure (PDB ID: 3LZG) has only 506 amino acids. Hence, the complete HA_Cal_ structure was predicted using the *ab initio* modeling strategy implemented in Robetta (Raman et al., 2009; Song et al., 2013). The sequences of HA_Cal_ and HA_Ind-2018_ (GQ280797 and QCP70896, respectively, in FASTA format) were retrieved from the NCBI databank (http://www.ncbi.nlm.nih.gov/) and utilized for homology modeling. The superimposition of both crystal and modeled HA_Ind-2018_ and HA_Cal_ structures is shown in Supplementary Figure S5.1 and Supplementary Figure S5.2. The modeled HA protein structures were analyzed by WHAT IF and SAVES (http://nihserver.mbi.ucla.edu/SAVES/) servers and were visualized by UCSF Chimera (v.1.15) software (Pettersen et al., 2004). The robustness of the generated models was ensured by the RAMPAGE server (Ramachandran map).

### 2.6 Receptor-binding site (RBS) analysis

Receptor-binding site analysis was performed on the HA_Ind_ protein to examine the mutation-mediated variation that emerged at the binding sites when compared to the HA_Cal_ protein. Wei Hu et al. reported the highly conserved receptor-binding domains of the HA protein of the influenza A (H1N1) virus (Hu, 2010), and their information was used while characterizing both HA_Ind_ and HA_Cal_ strains.

### 2.7 Epitope-binding site (EBS) analysis

The analysis of the EBS gains importance as it provides the hotspot for membrane fusion between the host and pathogens. Analysis of the conserved EBS is essential to understand its dominance over the recognition of the antibody. Apart from the canonical/native epitope sites, the identification of mutation-derived new epitope sites is also essential to explain the exact viral–host interaction during the membrane fusion mechanism. Epitope sites of both HA_Ind_ and HA_Cal_ proteins of the influenza A (H1N1) virus were analyzed in the SVMTriP web server (http://sysbio.unl.edu/SVMTriP) using default parameters to explain both the conserved EBS and the newly emerging EBS due to mutation. The potential antigenic sites in the HA sequence were examined using a string kernel-based support vector machine (SVM), SVMTriP (Yao et al., 2012). This SVM model calculates the similarity using the BLOSUM62 matrix for the tripeptides or trimers from the input sequences given in FASTA format. Finally, the predicted epitopes, within the default limit of 20 sites, are ranked according to their scores.

### 2.8 N-glycosylation site analysis

One of the most influential post-translational modifications is N-glycosylation, which affects antigenicity, biological activity, cell–cell interactions, protein solubility, protein folding, localization, and trafficking*.* Here, the N-glycosylation sites across functional domains of the HA protein are mapped to locate both known and mutation-derived new sites as well. The NetNGlyc 1.0 server (https://services.healthtech.dtu.dk/service.php?NetNGlyc-1.0) was used with default parameters to analyze the N-glycosylation sites that are conserved among the Indian isolates of influenza A (H1N1) viruses. The NetNGlyc 1.0 server predicts all possible sequence patterns, “N-X-S/T” (any amino acids except P at the X position) within HA protein sequences as potential N-glycosylation sites, based on an artificial neural network approach. The most probable N-X-S/T patterns with the highest percentage of occurrence are filled out using the cutoff value of 0.5. The locations of the predicted N-glycosylation sites in the monomer of the HA_Ind_ protein are numbered according to the full-length HA_Cal_ protein sequence.

### 2.9 Amino acid composition analysis

The ProtParam (https://web.expasy.org/protparam/) tool implemented in the ExPASy server is capable of predicting various physicochemical properties from the sequence, such as molecular weight, pI, amino acid composition, atomic composition, extinction coefficient, estimated half-life, instability index, aliphatic index, and grand average of hydropathicity (GRAVY) from the sequence. Here, variation in the amino acid composition of HA_Ind_ proteins reported during 2009–2018 is analyzed in comparison with the HA_Cal_ protein using ProtParam with default parameters to understand the genetic susceptibility and evolution of Indian isolates compared with the pandemic strain (A/California/04/2009).

### 2.10 Secondary structure prediction

A high degree of conformational plasticity may present a barrier to the development of beneficial antibodies. The GOR IV web server (https://npsa-prabi.ibcp.fr/cgi-bin/npsa_automat.pl?page=npsa_gor4.html) was used with default parameters to 1) understand the degree of conformational plasticity by analyzing the secondary structure (alpha helix, extended strand, and random coil) and also 2) further illustrate the variable and invariable structural changes in HA_Ind_ proteins reported during 2009–2018 compared with the selected HA_Cal_ protein.

### 2.11 Electrostatic potential (ESP) analysis

Electrostatic interactions (EIs) play a vital role in determining biomolecular functions. In particular, the EIs, which govern biomolecular sensing, are highly regulated by the nature of electrostatic potential. Hence, analysis of effective biomolecular sensing requires a thorough characterization of the distribution of ESP over the biomolecular surface boundaries. Here, the electrostatic charge distribution over the surface of both HA_Ind_ and HA_Cal_ proteins is calculated at an ionic strength of 0.15 M and visualized/analyzed using the Adaptive Poisson–Boltzmann Solver (APBS) plugin, integrated in VMD software (version 1.9.3). The ESP, represented as an isoelectrostatic potential map, depicts red and blue contours with values of −5.0 and +5.0 kBT/e^−^, respectively.

## 3 Results

### 3.1 HA sequence retrieval of Californian and selected Indian strains

About 512 HA_Ind_ protein sequences, reported during 2009–2018 from various geographical locations in India until October 2020, are available in the NCBI repository database under the subsection “Influenza virus.” We collected the available HA_Ind_ protein sequences in FASTA format (Supplementary File S1) and performed the BLAST search for the identification of reference strains, followed by MSA. It is interesting to observe that the HA_Cal_ protein of the A/California/04/2009 strain disclosed a very close relation with the 10 Indian isolates (given in Table 1 **)** reported from 2009 to 2018. Hence, these strains are selected for further comparative analyses to explore the evolution of HA_Ind_ proteins. The 3D structures of the HA_Ind_ protein are unavailable, whereas the crystal structures of the HA_Cal_ protein, available in PDB (for example, PDB ID: 3AL4, 3LZG, 3UBE, 3UBJ, 3UBN, 3UBQ, 3UYW, 3UYX, 3ZTN, 4JTV, 4JTX, 4JU0, 4M4Y, 5GJS, 5K9O, 5WKO, 6URM, and 6WJ1), lack a complete structure (Xu et al., 2010; Corti et al., 2011; Xuan et al., 2011; Xu et al., 2012; Hong et al., 2013; Zhang et al., 2013; Zhang et al., 2013; Joyce et al., 2016; Wang et al., 2016; Lang et al., 2017; Cheung et al., 2020; Wu et al., 2020). Due to the lack of a complete structure of the HA protein, we modeled the complete structure of both HA_Cal_ and HA_Ind-2018_ using the full-length sequence (566 aa) and compared it with the reported crystal structure (PDB ID: 3LZG). The superimposition of both crystal and modeled structures reveals similar architecture, as shown in Supplementary File S5. Hereafter, the three-dimensionally modeled structures of the entire sequence of HA_Cal_ and HA_Ind-2018_ are used for further comparative structural analyses.

### 3.2 Evolutionary relationship analysis

Knowledge on the extent of genetic reassortment, antigenic shifts, and drifts in HA surface proteins of the H1N1 influenza virus isolates reported in India has become an indispensable concept as it discloses the most important factors related to its virulence. By examining the evolution of sequences, we tried to highlight how the selective pressure on the viral protein changes over time, leading to alterations in antigenicity, which further discloses variation in host specificity toward their receptor. A total of 512 HA protein sequences reported from the Indian strains of H1N1 viruses circulated during 2009–2018 (HA_Ind_) were retrieved from the NCBI flu database (Supplementary File S1). At first, an exhaustive MSA was performed on these selected HA_Ind_ and HA_Cal_ sequences using ClustalW. In ClustalW, the sequences expressing variations due to mutation during the evolution of virus strains are aligned in accordance with the evolutionary distance and are further analyzed for phylogenetic relationships in a year-wise manner. A comparison of all HA_Ind_ proteins with reference to HA_Cal_ revealed the presence of new mutations as well. The MSA and phylogenetic tree (constructed using PAUP) revealed that HA_Ind_ proteins (evolved from 2009 to 2018, as given in Table 2) share a close evolutionary relationship with the HA_Cal_ protein (Supplementary File S2) and were chosen for further investigation.

### 3.3 Mutational analysis on HA_Cal_ and HA_Ind_ protein sequences

**FIGURE 1:**
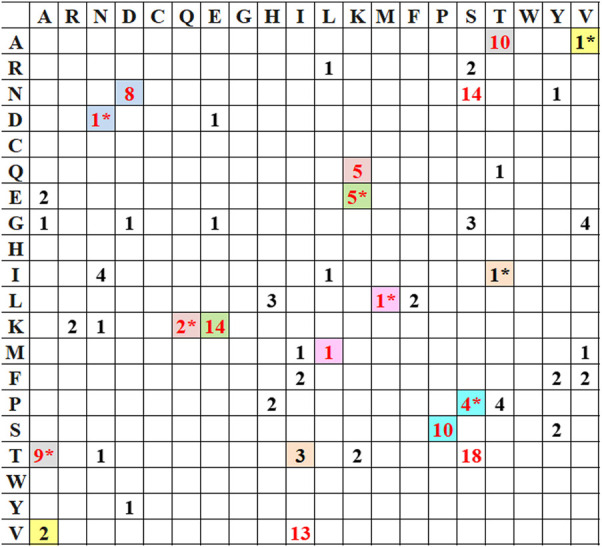
Frequency distribution of conceivable mutations (in numeric) empirically observed with HA_Ind_ strains compared with the reference HA_Cal_ strain. Similar colors in the plot depict similar mutations, but the order of mutation from one residue to another is differentiated with “*.”

Mutational analysis on the selected 10 HA_Ind_ sequences was performed in comparison with HA_Cal_ disclosed additional phenotypic variations at 84 positions, out of which 16 mutations gain importance as they share more conservation. Table 3 lists all the observed mutations in the selected HA_Ind_ proteins. In HA_Ind_, positions 114, 180, 273, 300, 516, 202, 468, 214, 220, 100, 338, and 391 (the grey highlighted positions in Table 3) have a greater probability of mutation than HA_Cal._ For a better understanding, the frequency (cumulative occurrence) of a mutation, in comparison with the reference HA_Cal_, is calculated and depicted in the frequency plot (Figure 1). The residues disclosing more than 50% of mutational occurrences are in red font. Similar colors in the plot depict similar mutations, but the order of mutation from one residue to another is differentiated with the “*” symbol. For instance, the frequency of mutation of proline to serine is observed 10 times in HA_Ind_ proteins, whereas the mutation of serine to proline in HA_Ind_ proteins is only observed four times.

**TABLE 3:** Mutations (bold fonts) in the HA_Ind_ proteins reported during 2009-2018 are compared with the HA_Cal_ protein (2009) and are highlighted in grey

Accession number	Strain name	Position of amino acids mutation in HA surface protein																											
		8	11	13	15	33	61	91	100	101	103	114	128	138	142	143	146	150	155	160	164	174	179	180	181	190	192	197	198
GQ280797	A/California/04/2009	L	T	A	A	V	L	S	P	S	D	D	F	S	N	H	N	T	H	S	N	S	S	K	S	V	V	H	H
AEM63482	A/Blore/NIV1196/2009	L	T	A	A	V	L	S	**S**	S	D	D	**L**	**G**	**I**	**L**	N	**P**	**L**	**T**	**I**	**P**	**T**	K	S	**A**	**G**	**P**	**L**
AIU46627	A/Khargone/293/2010	L	T	A	A	V	L	S	**S**	S	D	D	F	S	N	H	N	T	H	S	N	S	S	K	S	V	V	H	H
AEX63612	A/India/GWL01/2011	L	T	A	A	V	L	S	**S**	S	D	D	F	S	N	H	N	T	H	S	N	S	S	K	S	V	V	H	H
AJE62498	A/Delhi/057/2012	L	T	A	A	**G**	L	S	**S**	S	**N**	D	F	S	N	H	N	T	H	S	N	S	S	K	S	V	V	H	H
AKE37409	A/India/Alp135125/2013	**M**	T	A	A	V	L	S	**S**	**G**	D	D	F	S	N	H	N	T	H	**G**	N	S	S	K	S	V	V	H	H
ARG42801	A/Kerala/RGCB140815/2014	L	T	A	A	V	L	S	**S**	S	D	**N**	F	S	N	H	N	T	H	S	N	S	S	**Q**	S	V	V	H	H
ALK80385	A/India/DRDE_GWL719/2015	L	T	A	A	V	L	S	**S**	S	D	**N**	F	S	N	H	**D**	T	H	S	N	S	S	**Q**	S	V	V	H	H
ASJ82246	A/India/P167512/2016	L	T	**T**	A	V	**I**	S	**S**	**N**	D	**N**	F	S	N	H	N	T	H	S	N	S	**N**	**Q**	S	V	V	H	H
ASJ82235	A/India/P1722376/2017	L	**I**	**T**	**T**	V	L	S	**S**	**N**	D	**N**	F	S	N	H	N	T	H	S	N	S	**N**	**Q**	S	V	V	H	H
QCP70896	A/India/Che-1851811/2018	L	T	**T**	A	V	L	**R**	**S**	**N**	D	**N**	F	**N**	N	H	N	T	H	S	N	S	**N**	**Q**	**T**	V	V	H	H
Accession number	Strain name	Position of amino acids mutation in HA surface protein																											
		200	202	210	213	214	215	216	217	218	220	223	224	232	233	234	237	239	240	245	246	247	257	261	266	273	274	278	280
GQ280797	A/California/04/2009	S	S	Q	D	T	Y	V	F	V	S	Y	S	A	I	R	V	D	Q	N	Y	Y	I	A	V	A	M	A	S
AEM63482	A/Blore/NIV1196/2009	**P**	S	**K**	**N**	**A**	**F**	**F**	**L**	**G**	S	**S**	**R**	**E**	I	**K**	**M**	**G**	**K**	N	**F**	**S**	I	**E**	**G**	A	**L**	**V**	**P**
AIU46627	A/Khargone/293/2010	S	S	Q	D	**A**	Y	V	F	V	**T**	Y	S	A	I	R	V	D	Q	N	Y	Y	**V**	A	V	A	M	A	S
AEX63612	A/India/GWL01/2011	S	S	Q	D	**A**	Y	V	F	V	**T**	Y	S	A	I	R	V	D	Q	**I**	**N**	Y	I	A	V	A	M	A	S
AJE62498	A/Delhi/057/2012	S	S	Q	D	**A**	Y	V	F	V	**T**	Y	S	A	I	R	V	D	Q	N	Y	Y	I	A	V	A	M	A	S
AKE37409	A/India/Alp135125/2013	S	**T**	Q	D	T	Y	V	F	V	**T**	Y	S	A	I	R	V	D	Q	N	Y	Y	I	A	V	A	M	A	S
ARG42801	A/Kerala/RGCB140815/2014	S	**T**	Q	D	**A**	Y	V	F	V	**T**	Y	S	A	I	R	V	D	Q	N	Y	Y	I	A	V	**T**	M	A	S
ALK80385	A/India/DRDE_GWL719/2015	S	**T**	Q	D	**A**	Y	V	F	V	**T**	Y	S	A	I	R	V	D	Q	N	Y	Y	I	A	V	**T**	M	A	S
ASJ82246	A/India/P167512/2016	S	**T**	Q	D	**A**	Y	V	F	V	**T**	Y	S	A	**T**	R	V	D	Q	N	Y	Y	I	A	V	**T**	M	A	S
ASJ82235	A/India/P1722376/2017	S	**T**	Q	D	**A**	Y	V	F	V	**T**	Y	S	**G**	**T**	R	V	D	Q	N	Y	Y	I	A	V	**T**	M	A	S
QCP70896	A/India/Che-1851811/2018	**P**	**T**	Q	D	**A**	Y	V	F	V	**T**	Y	S	A	**T**	R	V	D	Q	N	Y	Y	I	A	V	**T**	M	A	S
Accession number	Strain name	Position of amino acids mutation in HA surface protein																											
		282	283	286	289	290	293	294	295	298	300	302	304	312	319	338	391	405	415	421	426	442	456	468	491	508	514	516	523
GQ280797	A/California/04/2009	I	I	D	V	H	N	T	T	T	K	A	N	I	K	I	E	T	N	I	K	L	D	S	T	E	R	E	E
AEM63482	A/Blore/NIV1196/2009	**F**	**F**	**N**	**F**	**P**	**I**	**P**	**P**	**P**	K	**V**	**T**	I	K	**V**	E	T	N	I	K	L	D	S	T	E	R	E	E
AIU46627	A/Khargone/293/2010	I	I	D	V	H	N	T	T	T	K	A	N	I	K	**V**	**K**	T	N	I	K	L	D	S	T	E	R	E	E
AEX63612	A/India/GWL01/2011	I	I	D	V	H	N	T	T	T	K	A	N	I	K	**V**	**K**	T	N	I	K	**R**	D	S	T	E	R	E	E
AJE62498	A/Delhi/057/2012	I	I	D	V	H	N	T	T	T	K	A	N	I	K	**V**	**K**	T	N	I	K	L	D	**N**	**A**	E	R	E	E
AKE37409	A/India/Alp135125/2013	I	I	D	V	H	N	T	T	T	K	A	N	I	K	**V**	**K**	T	N	I	K	L	**Y**	**N**	T	E	R	E	E
ARG42801	A/Kerala/RGCB140815/2014	I	I	D	V	H	N	T	T	T	**E**	A	N	**V**	K	**V**	**K**	T	N	I	K	L	D	**N**	T	**G**	R	**K**	E
ALK80385	A/India/DRDE_GWL719/2015	I	I	D	V	H	N	T	T	T	**E**	A	N	I	K	**V**	**K**	**Q**	**K**	I	**T**	L	D	**N**	T	E	R	**K**	E
ASJ82246	A/India/P167512/2016	I	I	D	V	H	N	T	T	T	**E**	A	N	I	K	**V**	**K**	T	N	I	K	L	D	**N**	T	E	R	**K**	E
ASJ82235	A/India/P1722376/2017	I	I	D	V	H	N	T	T	T	**E**	A	N	I	K	**V**	**K**	T	N	I	K	L	D	**N**	T	E	**K**	**K**	E
QCP70896	A/India/Che-1851811/2018	I	I	D	V	H	N	T	T	T	**E**	A	N	**V**	**T**	**V**	**K**	T	N	**M**	K	L	D	**N**	T	E	R	**K**	**D**

A year-wise analysis of mutational occurrence reported among the HA_Ind_ proteins from 2009 reveals that 1) all selected HA_Ind_ proteins possess mutations such as P100S, T214A, and I338V; 2) the mutations S220T and E391K are reported from 2010 onward; 3) the residues D114, K180, A273, K300, and E516 of HA_Cal_ remain conserved among the selected HA_Ind_ sequences reported until 2013 and the same sites disclosed mutations from 2014 onward such as D114N, K180Q, A273T, K300E, and E516K; 4) the mutations A13T, S101N, S179N, and I233T are reported since 2016; and 5) the mutations S202T and S468N are reported since 2013 and 2012, respectively. All these observations witness the occurrence of additional mutations that evolved over the successive period. It should be noted that the mutations of residues from T to A (T → A, S → N, D → N, K → Q, K → E, P → S, S → T, I → V, A → T, and E → K) play a significant role in the emerging diversity of HA_Ind_ proteins.

### 3.4 Receptor-binding site (RBS) in HA_Cal_ and HA_Ind_ protein sequences

Wei Hu et al. reported seven receptor-binding sites (RBS) that are highly conserved in the HA_Cal_ protein. The variations analyzed at the RBS of HA_Ind_ in comparison with the reported RBS of HA_Cal_ (Hu, 2010) are given in Table 4. The RBS of the HA_Ind_ strain (2009) is reported with 22 single amino acid mutations at the following positions: 1) RBS3 (S160T, N164I, and S173P), 2) RBS4 (H197P, H198L, S200P, and Q210K), 3) RBS5 (V218G, Y223S, S224R, and A232E), 4) RBS6 (V266G, M274L, A278V, S280P, I282F, and I283F), and 5) RBS7 (T294P, T295P, T298P, A302V, and N304T), in comparison with the HA_Cal_ protein. Few more mutations reported in HA_Ind_ strains during 2010–2018 are discussed in the following paragraphs.

**TABLE 4:** Mutations at the RBS of HA_Ind_ proteins (reported during 2009–2018) with reference to HA_Cal_ are indicated in bold fonts.

**Accession number**		**Strain’s name**	**Receptor-binding site (RBS)**					
			**RBS1 (22–40)**		**RBS2 (80–98)**		**RBS3 (159–176)**	
GQ280797		A/California/04/2009	IGYHANNSTDTVDTVLEKN		GNPECESLSTASSWSYIVE		KSFYKNLIWLVKKGNSYP	
AEM63482		A/Blore/NIV1196/2009	IGYHANNSTDTVDTVLEKN		GNPECESLSTASSWSYIVE		KTFYKILIWLVKKGNPYP	
AIU46627		A/Khargone/293/2010 2	IGYHANNSTDTVDTVLEKN		GNPECESLSTASSWSYIVE		KSFYKNLIWLVKKGNSYP	
AEX63612		A/India/GWL01/2011 20	IGYHANNSTDTVDTVLEKN		GNPECESLSTASSWSYIVE		KSFYKNLIWLVKKGNSYP	
AJE62498		A/Delhi/057/2012	IGYHANNSTDTGDTVLEKN		GNPECESLSTASSWSYIVE		KGFYKNLIWLVKKGNSYP	
AKE37409		A/India/Alp135125/2013	IGYHANNSTDTVDTVLEKN		GNPECESLSTASSWSYIVE		KSFYKNLIWLVKKGNSYP	
ARG42801		A/Kerala/RGCB140815/2014	IGYHANNSTDTVDTVLEKN		GNPECESLSTASSWSYIVE		KSFYKNLIWLVKKGNSYP	
ALK80385		A/India/DRDE_GWL719/2015	IGYHANNSTDTVDTVLEKN		GNPECESLSTASSWSYIVE		KSFYKNLIWLVKKGNSYP	
ASJ82246		A/India/P167512/2016	IGYHANNSTDTVDTVLEKN		GNPECESLSTASSWSYIVE		KSFYKNLIWLVKKGNSYP	
ASJ82235		A/India/P1722376/2017	IGYHANNSTDTVDTVLEKN		GNPECESLSTASSWSYIVE		KSFYKNLIWLVKKGNSYP	
QCP70896		A/India/Che-1851811/2018	IGYHANNSTDTVDTVLEKN		GNPECESLSTA**R**SWSYIVE		KSFYKNLIWLVKKGNSYP	

**Accession number**	**Strain’s name**		**Receptor-binding site (RBS)**					
			**RBS4 (195–212)**	**RBS5 (218–233)**		**RBS6 (263–286)**		**RBS7 (294–304)**
GQ280797	A/California/04/2009		GIHHPSTSADQQSLYQNA	VGSSRYSKKFKPEIAI		GNLVVPRYAFAMERNAGSGIIISD		TTCQTPKGAIN
AEM63482	A/Blore/NIV1196/2009		GI**PL**P**P**TSADQQSLY**K**NA	**G**GSSR**SR**KKFKPEI**E**I		GNLGVPRYAFALERNVGPGFFISD		**PP**CQ**P**PKG**V**I**T**
AIU46627	A/Khargone/293/2010 2		GIHHPSTSADQQSLYQNA	VG**T**SRYSKKFKPEIAI		GNLVVPRYAFAMERNAGSGIIISD		TTCQTPKGAIN
AEX63612	A/India/GWL01/2011 20		GIHHPSTSADQQSLYQNA	VG**T**SRYSKKFKPEIAI		GNLVVPRYAFAMERNAGSGIIISD		TTCQTPKGAIN
AJE62498	A/Delhi/057/2012		GIHHPSTSADQQSLYQNA	VG**T**SRYSKKFKPEIAI		GNLVVPRYAFAMERNAGSGIIISD		TTCQTPKGAIN
AKE37409	A/India/Alp135125/2013		GIHHPST**T**ADQQSLYQNA	VG**T**SRYSKKFKPEIAI		GNLVVPRYAFAMERNAGSGIIISD		TTCQTPKGAIN
ARG42801	A/Kerala/RGCB140815/2014		GIHHPST**T**ADQQSLYQNA	VG**T**SRYSKKFKPEIAI		GNLVVPRYAF**T**MERNAGSGIIISD		TTCQTP**E**GAIN
ALK80385	A/India/DRDE_GWL719/2015		GIHHPST**T**ADQQSLYQNA	VG**T**SRYSKKFKPEIAI		GNLVVPRYAF**T**MERNAGSGIIISD		TTCQTP**E**GAIN
ASJ82246	A/India/P167512/2016		GIHHPST**T**ADQQSLYQNA	VG**T**SRYSKKFKPEIA**T**		GNLVVPRYAF**T**MERNAGSGIIISD		TTCQTP**E**GAIN
ASJ82235	A/India/P1722376/2017		GIHHPST**T**ADQQSLYQNA	VG**T**SRYSKKFKPEI**GT**		GNLVVPRYAF**T**MERNAGSGIIISD		TTCQTP**E**GAIN
QCP70896	A/India/Che-1851811/2018		GIHHP**P**T**T**ADQQSLYQNA	VG**T**SRYSKKFKPEIA**T**		GNLVVPRYAF**T**MERNAGSGIIISD		TTCQTP**E**GAIN

**FIGURE 2:**
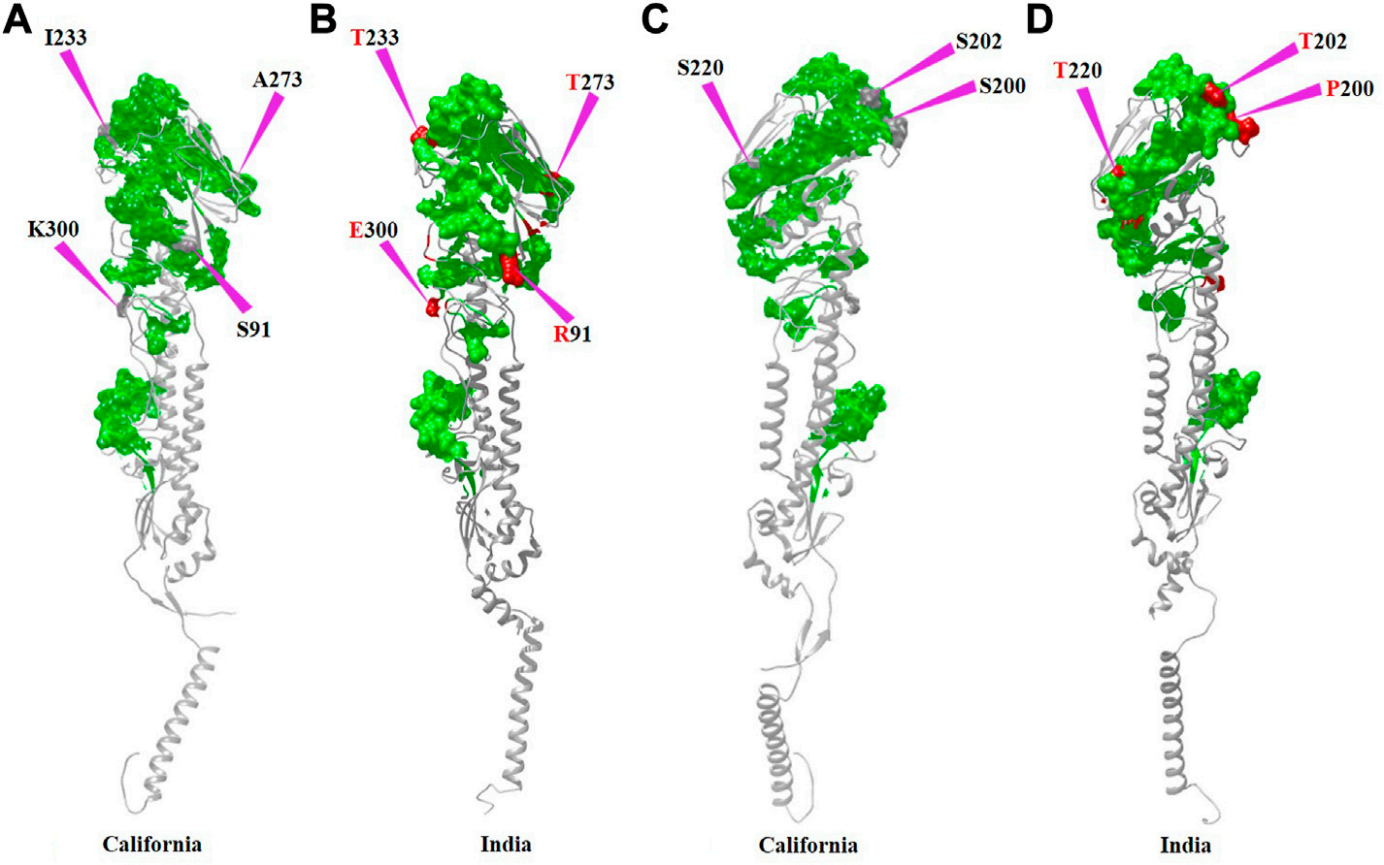
Receptor-binding sites of HA_Cal_ (green surface) are depicted in **(A,C)**, whereas those of HA_Ind-2018_ are shown in **(B,D)**. Additional mutations observed in HA_Ind_ are shown in the red surface and also indicated by purple arrows. To depict the mutational sites clearly, both front **(A,B)** and back **(C,D)** views of HA proteins are presented.

RBS1, RBS2, and RBS3 reveal mutations such as V33G (in 2012), S91R (in 2018), and S160G (2012), respectively. In RBS4, the mutation S202T that emerged in 2013 was conserved among the strains reported in successive years. RBS4 also witnessed an additional mutation (S200P) in the 2018 strain. In RBS5, all strains have inherited the A220T mutation along with few more such as 1) I233T in 2016, 2) A232G and I233T in 2017, and 3) I233T in 2018. In RBS6 and RBS7, mutations such as A273T and K300E were identified between 2014 and 2018. Specifically, the 2018 strain that emerged with seven mutations at the RBS (Figure 2) may imply that HA_Ind_ is more prone to mutation than HA_Cal_.

In general, except for the 2009 strain, the RBS analysis reveals that the HA_Ind_ strains circulated during 2010–2018 were significantly conserved except for the few aforementioned mutations. Despite the observed significant conservation at the sequence level, the emerging single mutation posed a challenge to the inhibitors in sensing the receptor-binding sites and hence prompted the scientific community to design sequence-specific receptor-binding agents for further inhibition.

#### 3.5 Epitope-binding site (EBS) in HA_Cal_ and HA_Ind_ protein sequences

Epitope mapping is critical in the development of vaccines or therapeutic monoclonal antibodies as it offers information on the mechanism of action. In the current study, the epitope-binding domains were analyzed using the SVMTriP web server, and the predicted epitope segments in the HA_Ind_ protein sequences are given in Supplementary Table S3 (Supplementary File S3) along with their rank and score. The analysis reveals about 10 antigenic sites when compared to the reference HA_Cal_ protein. Of these 10 antigenic sites, C-EBS1, C-EBS2, C-EBS8, and C-EBS10 (amino acid positions from 26–45, 66–85, 341–360, and 446–465, respectively) were also conserved in the Indian isolates during 2009–2018. C-EBS3 is not identified in Indian strains. C-EBS9 is reported only in 2009 strains, and in contrast, C-EBS6 is reported for all Indian strains except the 2009 strain. The sites C-EBS4 and C-EBS5 are also conserved but not reported in the 2009, 2015, and 2018 strains. C-EBS7 is observed only with 40% of occurrence.

**FIGURE 3:**
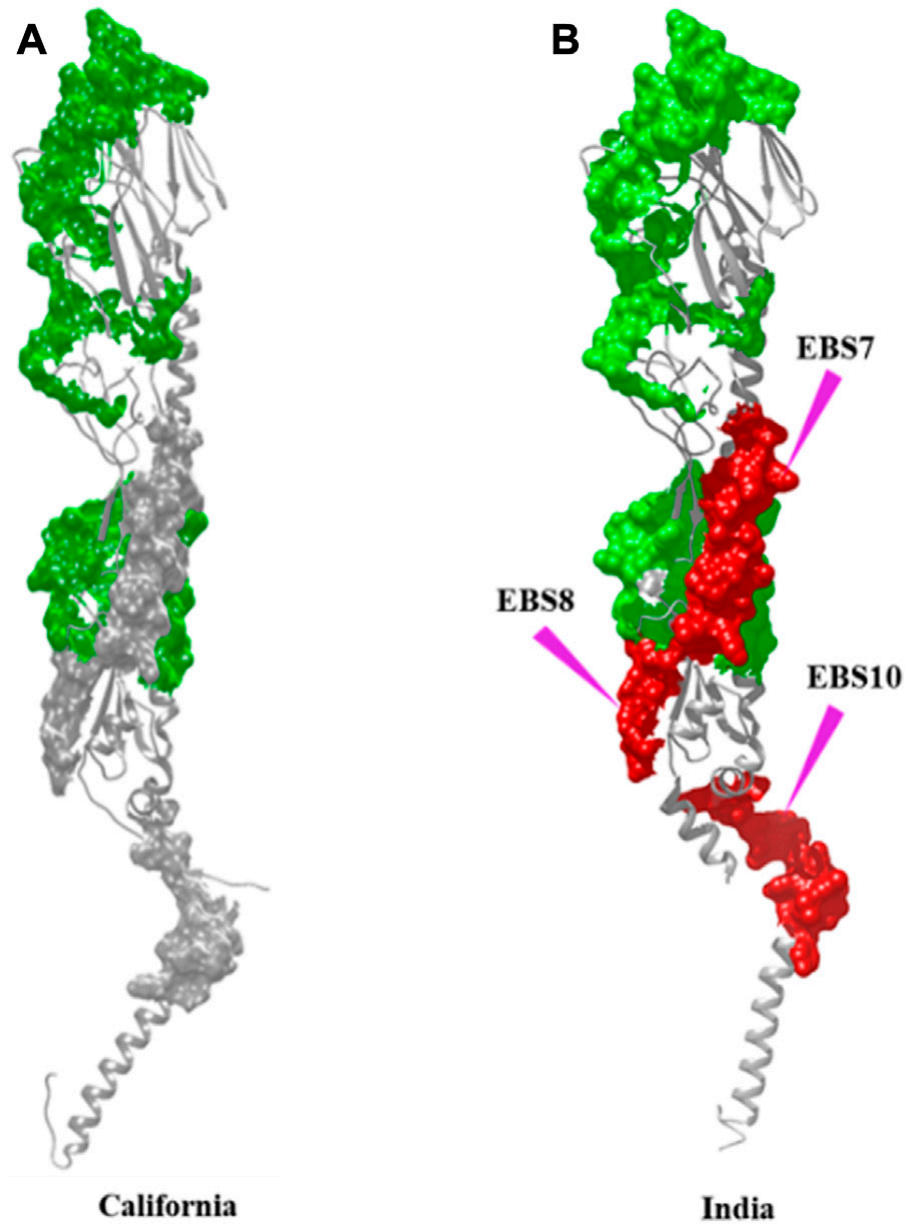
Antigenic sites, observed in common with both HA_Cal_ **(A)** and HA_Ind_ **(B)** strains, are shown in the green surface. Additional antigenic sites observed specific to HA_Ind_ proteins (2009–2018) are shown in the red surface. The sites of mutation are indicated by purple arrows.

In addition to the reported 10 C-EBSs, SVMTriP also identified 10 potential EBS (at residue positions 110–129, 146–165, 242–261, 317–336, 366–385, 386–405, 407–426, 408–427, and 515–534, and hereafter will be referred to as I-EBS) exclusively in HA_Ind_ proteins (Table 5). These predicted antigenic sites were analyzed using the SVMTriP tool and are listed according to the predicted score, rank, and positions of the EBS (Supplementary File S3). The sequence positions 1) 366–385 (YGYHHQNEQGSGYAADLKST) and 2) 386–405 (QNAIDKITNKVNSVIEKMNT**)** are identified as conserved I-EBS in 2010–2018, while sequences at 3) 515–534 (EKIDGVKLESTRIYQILAIY) are conserved I-EBS in 2014–2017 Indian isolates (Supplementary File S3). The results suggest that mutational events trigger more antigenic sites. The newly identified antigenic sites such as I-EBS7, I-EBS8, and I-EBS10 (Figure 3) are anticipated to provide more interacting sites in the target, which would eventually fine-tune the process of therapeutic drug/vaccine design.

**TABLE 5:** Top 10 antigenic sites identified in HA_Ind_ proteins using the SVMTriP server.

Top 10 epitope-binding sites identified in HA_Ind_ proteins					
No.	Rank	Position	Epitope-binding sites (hereafter referred to as I-EBS)		SVMTriP score
1	1	136–155	I-EBS3	KTSSWPNHDSNKGVTAACPH	1
2	2	26–45	I-EBS1	ANNSTDTVDTVLEKNVTVTH	0.997
3	3	366–385	I-EBS7	YGYHHQNEQGSGYAADLKST	0.996
4	3	341–360	I-EBS6	IQSRGLFGAIAGFIEGGWTG	0.996
5	3	276–295	I-EBS5	RNAGSGIIISDTPVHDCNTT	0.996
6	4	66–85	I-EBS2	PLHLGKCNIAGWILGNPECE	0.995
7	5	386–405	I-EBS8	QNAIDKITNKVNSVIEKMNT	0.98
8	6	446–465	I-EBS9	LENERTLDYHDSNVKNLYEK	0.858–0.994
9	7	160–179	I-EBS4	(G)SFYKNLIWLVKKGNSYPKLS(N)	0.869
10	8	515–534	I-EBS10	EKIDGVKLESTRIYQILAIY	0.292–0.426

#### 3.6 Prediction of N-glycosylation sites in HA_Cal_ and HA_Ind_ protein sequences

**FIGURE 4:**
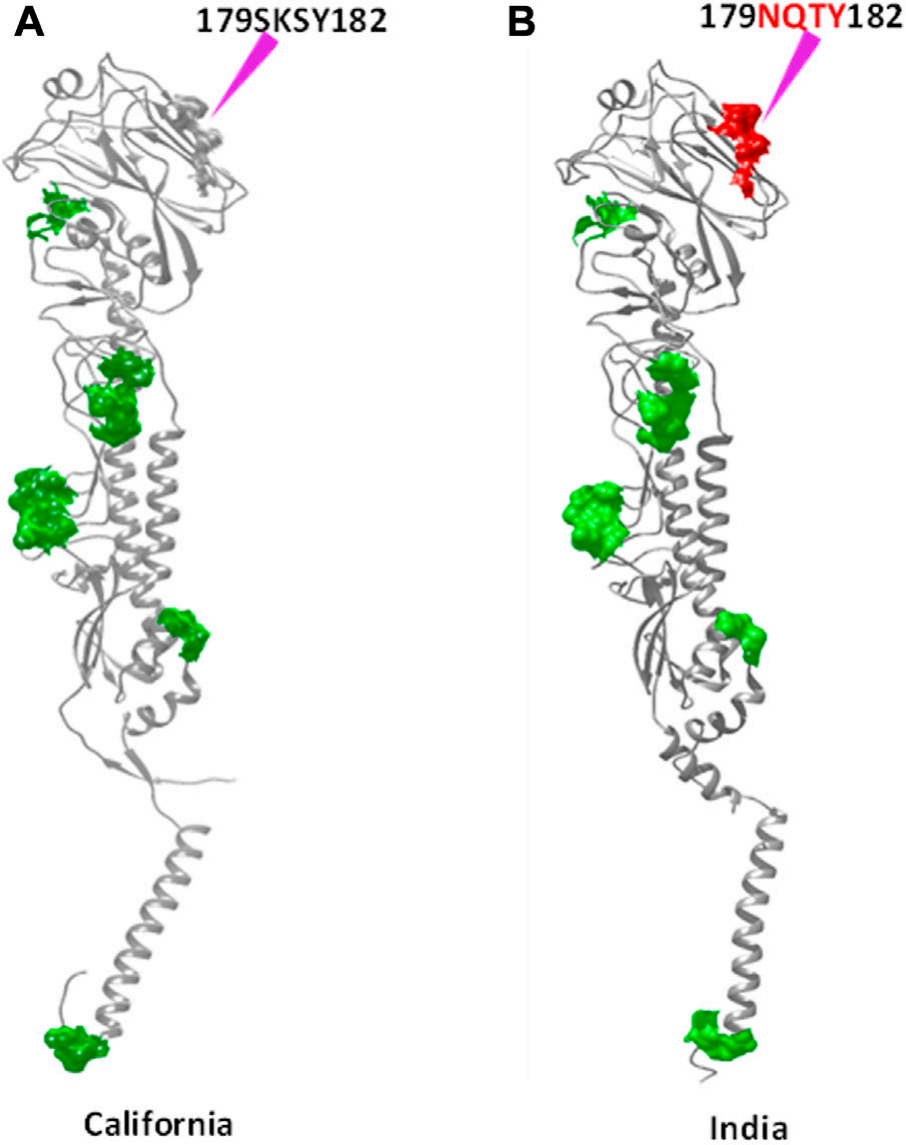
N-glycosylation sites observed in common with both HA_Cal_ **(A)** and HA_Ind_ **(B)** strains are shown in the green surface. The red surface indicates the newly formed N-glycosylation site due to mutation. The sites of mutation are indicated by purple arrows.

The attachment and release of viruses from their host cells exploit the phenomenon of glycosylation. For example, the N-glycosylation of the HA surface protein allows the pathogen to escape from the host’s defense mechanism through co-evolving with the host protein and eventually identifying the host receptor for further fusion. Hence, N-glycosylation sites are crucial in determining the H1N1 host binding and release factors, which subsequently determine the fate of virus infection in the host as well. In line with this importance, N-glycosylation sites were predicted in HA_Ind_ strains reported during 2009–2018 using NetNGlyc 1.0 v and are shown in Figure 4. The HA_Cal_ protein possesses about eight N-glycosylation sites, namely, 27NNST30, 28NSTD31, 40NVTV43, 104NGTC107, 293NNTC296, 304NTSL307, 498NGTY501, and 557NGSL560 (here, each N-glycosylation site is referred to with the starting and ending positions of amino acids).

As disclosed by HA_Cal_, the selected HA_Ind_ strains also disclose all these N-glycosylation sites except the Indian A/Blore/NIV1196/2009 strain (Table 6), which lacks 293NNTC296 and 304NTSL307 N-glycosylation sites. Along with these reported nine N-glycosylation sites, the HA_Ind_ strains reported during 2016–2018 reported an additional N-glycosylation site, 179NQSY182. A clear observation demonstrated that the amino acid 179SKSY182 of the HA_Cal_ protein was conserved in the HA_Ind_ strains reported from 2009 to 2013, and by the mutation K180Q (reported in 2014–2015), the amino acid segment 179SQSY182 evolved as a precursor to the identified 179NQSY182 N-glycosylation site (in which S is mutated into one of the active site forming residues, N) in the HA_Ind_ sequences reported in the subsequent years (2016–2018).

**TABLE 6:** Predicted N-glycosylation sites of HA_Ind_ compared with the reference HA_Cal_ proteins. The newly emerged N-glycosylation sites (amino acids in 179–182 positions), specific to HA_Ind_ strains, are shown in bold.

**Accession number**	**Strain’s name**	**Amino acid positions**								
		**27–30**	**28–31**	**40–43**	**104–107**	**179–182**	**293–296**	**304–307**	**498–501**	**557–560**
GQ280797	A/California/04/2009	NNST	NSTD	NVTV	NGTC	SKSY	NTTC	NTSL	NGTY	NGSL
AEM63482	A/Blore/NIV1196/2009	NNST	NSTD	NVTV	NGTC	TKSY	IPPC	TTSL	NGTY	NGSL
AIU46627	A/Khargone/293/2010 2	NNST	NSTD	NVTV	NGTC	SKSY	NTTC	NTSL	NGTY	NGSL
AEX63612	A/India/GWL01/2011 20	NNST	NSTD	NVTV	NGTC	SKSY	NTTC	NTSL	NGTY	NGSL
AJE62498	A/Delhi/057/2012	NNST	NSTD	NVTV	NGTC	SKSY	NTTC	NTSL	NGTY	NGSL
AKE37409	A/India/Alp135125/2013	NNST	NSTD	NVTV	NGTC	SKSY	NTTC	NTSL	NGTY	NGSL
ARG42801	A/Kerala/RGCB140815/2014	NNST	NSTD	NVTV	NGTC	SQSY	NTTC	NTSL	NGTY	NGSL
ALK80385	A/India/DRDE_GWL719/2015	NNST	NSTD	NVTV	NGTC	SQSY	NTTC	NTSL	NGTY	NGSL
ASJ82246	A/India/P167512/2016	NNST	NSTD	NVTV	NGTC	NQSY	NTTC	NTSL	NGTY	NGSL
ASJ82235	A/India/P1722376/2017	NNST	NSTD	NVTV	NGTC	NQSY	NTTC	NTSL	NGTY	NGSL
QCP70896	A/India/Che-1851811/2018	NNST	NSTD	NVTV	NGTC	NQTY	NTTC	NTSL	NGTY	NGSL

It is also vital to ensure the structurally stable evolution of Indian strains by retaining the characteristic hydrophobic/hydrophilic interactions despite the encountered mutations. Therefore, a detailed study about the 3D structure of viral proteins along with physico-chemical characterization would be useful to understand the changes in viral activity attributed to the changes at the sequence level.

#### 3.7 Amino acid composition of HA_Cal_ and HA_Ind_ protein sequences

**FIGURE 5:**
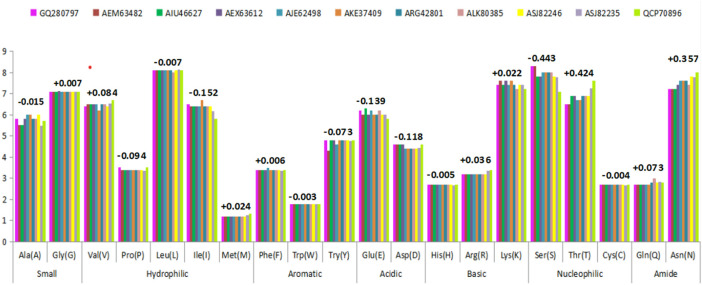
Variations observed in amino acid composition due to mutational events in the HA_Ind_ protein sequence circulated during 2009–2018 against HA_Cal_. The numerical values (either with + or – sign) indicate the average variations of mutational events.

The amino acid compositional variation of HA_Ind_ proteins reported during 2009–2018 was compared with that of HA_Cal_ using the ProtParam server (Figure 5) to understand the impact of the mutational effect on the number of compositional amino acids toward the conformational stability of HA proteins (Supplementary File S4). A comparison of the statistical occurrence of each amino acid in HA_Ind_ with HA_Cal_ revealed a few interesting observations. For example, about 32 serine amino acids of the HA_Cal_ strain mutated into threonine and asparagine in HA_Ind_ with a statistical occurrence of 18 and 14, respectively. This shows that the propensity of serine getting mutated into threonine and asparagine is more prevalent in Indian strains. The observation of S mutating into T (a crucial amino acid in forming the active site of an enzyme) and N (one of the critical factors reported to regulate viral replication) (Lee et al., 2019) has biological significance. In particular, the observation of the new N-glycosylation site 179NQTY182 (which also forms a part of EBS4 observed at 160 (G) SFYKNLIWLVKKGNSYPKLS (N) 179 (Supplementary File S3), reported in the Indian strains from 2016 to 2018, is one such example. It is also vital to disclose the intermediate stages of mutation (S to N) from 179SKSY182 to 179SQSY182 and, finally, to 179NQSY182 over the studied period (Table 6). Another example of S mutated as T, resulting in RBS4 (195GIHHPSTSADQQSLYQNA212 to 195GIHHPSTTADQQSLYQNA212) and RBS5 (218VGSSRYSKKFKPEIAI233 to 218VGTSRYSKKFKPEIAI233), is shown in Table 4.

#### 3.8 Predicted secondary structure of HA_Cal_ and HA_Ind_ proteins

**FIGURE 6:**
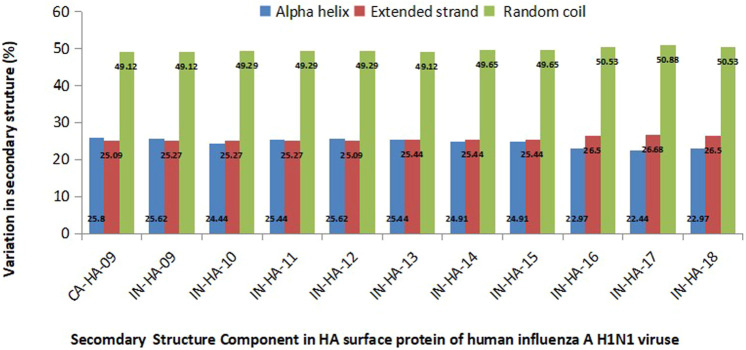
Variations in the composition of secondary structures in both HA_Cal_ and HA_Ind_ strains. The percentage of alpha helices (blue bars), extended strands (red bars), and random coils (green bars) is depicted (please also refer Table 2).

Secondary structures of HA_Cal_ and HA_Ind_ sequences, pertaining to their structural stability, are analyzed using the GOR IV web server. Figure 6 depicts the compactness of the 3D structure of HA stains in terms of the fraction of residues forming the structural elements, particularly helix, sheet, and random coil. Analysis of the composition of the secondary structure in all HA_Ind_ sequences revealed the prevalence of a high proportion (50%) of random coils when compared to the helix and extended sheets (which equally share 25% each). It should be noted here that the equal contribution of both helix and extended sheets is retained in the HA_Ind_ strains until 2015. In the HA_Ind_ strains reported from 2016 to 2018, the overall helical components are reduced by 2%, and accordingly, the occurrence of both extended sheets and random coils is increased. Such an observation is witnessed by the transformation of a few helices into extended sheets and random coils (for example, the amino acid segments 9–12, 230–234, and 236–241).

Overall, the present analysis indicates the high occurrence of random coils in all selected HA_Ind_ strains as one of the potentially unique characteristics of HA strains, which lowers the structural compactness (along with additional contributions from the helices to extended sheets and random coils). Such increased random coil segments enhance the structure flexibility, thereby promoting an effective interaction with other essential components of the host.

#### 3.9 Electrostatic potential (ESP) of HA_Cal_ and HA_Ind_ proteins

**FIGURE 7:**
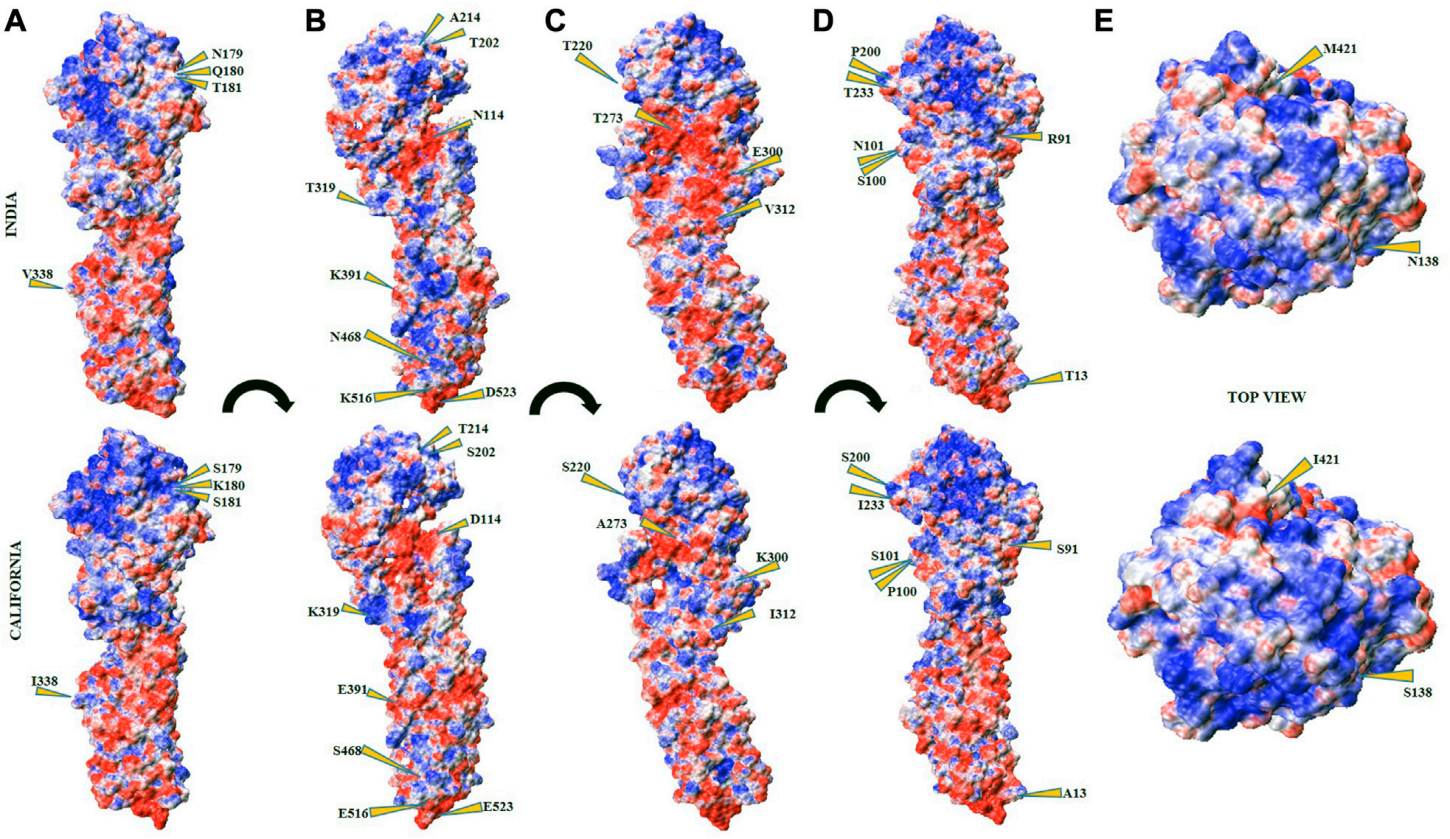
Electrostatic potential surface (ESPS) of HA_Ind_ (top panel) and HA_Cal_ (bottom panel) proteins. The subsets **(A–D)** depict different orientations from the front view of HA protein surfaces in terms of 90° with respect to the long axis passing from HA1 to HA2 domains. The subsets **(E)** depict the top view of HA from the HA1 domain. The positive, negative, and neutral ESPS are depicted in blue, red, and white surfaces, respectively.

The host cell defense mechanism is highly sensitive to the physicochemical nature of the interacting viral particle, and the emerging mutations perturb their sensing mechanism. At the molecular level, explicitly, the EIs take a lead role in establishing strong complex formation. The electrostatic potential surfaces (ESPSs) of the HA proteins from both Indian (2018) and California strains are compared and contracted to better understand the potential of the Indian strain (Figure 7). Both electropositive and negative potential sites are shown in blue and red surfaces, respectively, along with the near-neutral residues as white surfaces. The mutated residues are labeled and indicated using yellow arrows. The ESP map, depicting the distribution of both positive and negative ESPSs of HA proteins, was generated using the Adaptive Poisson–Boltzmann Solver (APBS) to compare and contrast the electrostatic features of HA_Ind-2018_ and HA_Cal_ proteins. Along with the ESPS, the effect of mutations on the solvent accessibility of both HA_Ind-2018_ and HA_Cal_ proteins was also analyzed (Figure 7).

Table 7 lists all mutated RBD residues in both HA_Ind_ and HA_Cal_ proteins. From the examination of the ESPS of both strains, it could be speculated that the HA_Ind_ protein could get attached to the receptor more efficiently due to the emergence of potential electrostatic interactions. Some of the mutations observed at the RBD of the HA_Ind_ protein are predicted to affect the antibody neutralization mechanism either by introducing conformational changes locally in the HA protein due to S91R, S200P, S202T, S220T, I233T, A273T, and K300E mutations (Amin et al., 2020; Gan et al., 2022; Jawad et al., 2022) or by altering its surface charge distribution due to D114N, K180Q, K300E, K319T, and E391K mutations. Such significant redistribution of the ESPS promotes increased resistivity against known therapeutics when compared to the HA_Cal_ strain.

**TABLE 7:** List of mutated residues and their locations in both HA_Cal_ and HA_Ind_ protein structures. The RBD residues are in bold, and the mutated residues (from charged to uncharged and *vice versa*) are indicated by *.

Amino acid position	Residues		Domain/motif
	HA_Cal_	HA_Ind-2018_	
13	Ala	Thr	HA1/β-strand
**91**	Ser (*)	**Arg (+)**	**HA1/β-hairpin**
100	Pro	Ser	HA1/β-turn
101	Ser	Asn	HA1/β-turn
114	Asp (−)	Asn (*)	HA1/β-turn
138	Ser	Asn	HA1/β-turn
179	Ser	Asn	HA1/β-strand
180	Lys (+)	Gln (*)	HA1/β-strand
181	Ser	Thr	HA1/β-strand
**200**	**Ser**	**Pro**	**HA1/β-strand**
**202**	**Ser**	**Thr**	**HA1/α-helix**
214	Thr	Ala	HA1/β-turn
**220**	**Ser**	**Thr**	**HA1/β-strand**
**233**	**Ilu**	**Thr**	**HA1/α-helix**
**273**	**Ala**	**Thr**	**HA1/β-strand**
**300**	**Lys (+)**	**Glu (−)**	**HA1/β-turn**
312	Ilu	Val	HA1/β-strand
319	Lys (+)	Thr (*)	HA1/β-strand
338	Ilu	Val	HA1/β-turn
391	Glu (−)	Lys (+)	HA2/α-helix
421	Ilu	Met	HA2/α-helix
468	Ser	Asn	HA2/α-helix
516	Glu (−)	Lys (+)	HA2/β-turn
523	Glu (−)	Asp (−)	HA2/β-strand

## 4 Discussions

The HA protein of the influenza A (H1N1) virus is known to play a significant role in the entry of viruses into the host and their pathogenicity as well. An “effective HA target-based vaccine/drug” has become a pressing need for society. The complexity in designing HA inhibitors arose due to several factors, including the higher rate of missense/point mutations. The H1N1 strain, A/California/04/2009, is the closest neighbor of all strains reported in India during the 2009 pandemic. A methodical analysis of the HA proteins of Indian strains from 2009 to 2018 was performed and compared with that of the A/California/04/2009 strain. The HA_Ind_ strains, reported with more specific mutations at a higher rate, emerged with enhanced virulence (Tharakaraman and Sasisekharan, 2015) and also became resistant to antiviral drugs such as oseltamivir, zanamivir, and peramivir (Parida et al., 2016; Tandel et al., 2018). These viruses with frequent reassortment at the sequence level evolved as more virulent than the previous seasonal H1N1 viruses (Baillie and Digard, 2013; Su et al., 2015; Luo et al., 2018a) and acquired better abilities to infect humans, which caused worse outbreaks (Luo et al., 2018b).

### 4.1 Evolutionary relation between HA proteins

Here, we present a systematic analysis of the HA proteins of H1N1 to understand the adaptation and divergence among Indian strains due to frequent mutational events. Particularly, the analyses focused on the impact of missense mutations on receptor-binding domains, antigenic site alteration, N-glycosylation site prediction, amino acid compositional variability, and associated variability in secondary structure*.* In line with this, the present analysis also exclusively emphasizes on Indian strains and compares them with a recognized reference pandemic strain to understand the challenges behind the failure of successful medication in the Indian context. Accordingly, about 512 Indian strains were retrieved along with the A/California/04/2009 strain from the flu database of the NCBI reported during 2009–2018 and were analyzed by MSA to explore the evolutionarily conserved genetic regions. Site-specific variations observed in the aligned sequences, reflecting the rate of mutations or degree of evolution, were further analyzed to figure out the evolutionarily conserved regions with reference to the strain A/California/04/2009. A one-to-one relation between the aligned sequences was visualized using PAUP-generated phylogenetic tree, which displayed the closest evolutionary relationship between Indian and Californian strains of the influenza A (H1N1) virus (Supplementary File S2). Variations in sequences due to missense mutations at various positions play a vital role in altering the structure and function of different domains of the HA protein, such as the receptor-binding domain, epitope-binding domain, and N-glycosylation site.

Alignment of the amino acid sequences of HA_Ind_ reported during 2009–2018 showed about 84 amino acid substitutions when compared to the reference HA_Cal_ protein. About 24 substitutions were observed in HA_Ind-2018_, in which 16 were highly concerned (Table 3). Analyses reveal that among the 16 mutations, seven mutations were found in the receptor-binding sites (Table 4), four were in antigenic sites (Table 5), and three were involved in the formation of N-glycosylation sites (Table 6). The HA_Ind_ strains are characterized by the mutations P100S, T214A, S220T, I338V, and E391K, i.e., possible beneficiary mutations that got fixed in the strains reported during 2009–2018 (Table 3). The literature suggests that T214A substitution in HA genes decreases the binding affinity with the host receptor (de Vries et al., 2013). We observed six new amino acid mutations (S91R, S138N, S200P, K319T, I421M, and E523D) in HA_Ind-2018_. The mutations S91R and S200P were found to be unique in HA_Ind-2018_, and these substitutions were abundant in the complete HA population (in 2018) compared to the pandemic HA_Cal_. The substitutions A13T, S101N, D114N, I312V, S468N, and E516K were also observed in HA_Ind_ and are also reflected in the recent studies (Biswas et al., 2019; Prasad et al., 2020; Siddiqui et al., 2020). In accordance with our results, another research group carried out a mutational examination of H1N1 with random samples and observed that viruses circulated during 2017 have 18 detected substitutions in HA_Ind_ (Jones et al., 2019). They also reported I233T, S179N, S181T, and I312V as new substitutions, among which S181T and I312V were presented as unique mutations in HA_Ind_ isolates (Jones et al., 2019). Interestingly, we did not find I312V in 2017. The observed amino acid substitutions (S91, S200, S202, A214, and I233) have been found in receptor-binding sites envisaged to vary during the adaptation process to α2-6-linked sialic acid receptors in humans (Maines et al., 2009). The I223T amino acid substitution is linked with increased binding affinity to human α2-6-linked sialic acid receptors (Al Khatib et al., 2019). Substitutions S200P and S202T are responsible for enhanced receptor-binding avidity by altering the receptor-binding affinity, whereas the A214T substitution is linked to the decreased binding avidity (de Vries et al., 2013). A previous study suggested that S202T is one of the responsible substitutions involved in increased mortality and morbidity (Adam et al., 2019). Studies also support our observations that mutations of HA like P100S, T214A, S220T, I338V, and E391K are conserved mutations specific to the dominant variant(s) of influenza A (H1N1) viruses during post-pandemic circulation in India (Morlighem et al., 2011; Jones et al., 2019; Siddiqui et al., 2020). It is also evident from research that the substitutions S181T and I312V in HA could lead to altered glycan specificity (Jones et al., 2019). The substitution K180Q triggers conformational variation in ligand binding, which might trigger the failure of specific ligand-binding properties as well (Jones et al., 2019). The mutation S179N, associated with glycosylation, is responsible for the increased pathogenicity of the viral particle by preventing the antigenic sites of immune recognition (Al Khatib et al., 2019).

### 4.2 Sequence and functional analysis of EBS, RBS, and N-glycosylation sites

Out of 84 mutational sites, about 12 most probable conserved mutational sites at amino acid positions 100, 114, 180, 202, 214, 220, 273, 300, 338, 391, 468, and 516 have been observed in the last five consecutive years (Table 3). The HA_Cal_ protein possesses seven characteristic receptor-binding sites (Hu, 2010) and has been compared against all HA_Ind_ strains. Indian strains expressed mutations mainly like serine-to-threonine, alanine-to-threonine, and lysine-to-glutamine at various binding sites (RBS 4–7) over a period of time, i.e., mutations S220T (at RBS 5), S202T (at RBS 4), A273T (at RBS 6), and K300E (at RBS7) were reported since 2010, 2013, 2014, and 2018, respectively (Table 4). The results suggest that these mutations may trigger the alteration in the RBD and become resistant to available therapeutic options. In comparison, all HA_Ind_ proteins emerge with more point mutations than the selected HA_Cal_, which play a significant role to evade from the known immune defense mechanism and further become life-threatening as well. Epitope mapping gains prime importance in the design of therapeutic monoclonal antibodies or vaccines, and any sequence-level mutations at the antigenic epitope sites hinder or delay the design of effective novel vaccines. In line with this, the studied Indian strains, which revealed significant mutations in the epitope-binding domains of HA proteins (Supplementary File S4), also delayed the successful identification of a vaccine for all Indian strains. Hence, faster evolution of the epitope-binding domain renders more complexity in the eradication process of the influenza A (H1N1) virus. Similar to epitope-binding sites, variations at N-glycosylation sites also increase complexity in the design of inhibitors (Zhang et al., 2004; Wei et al., 2010). Mutations in the glycosylation sites aid the HA protein (Table 6) to co-evolve with the host protein for the successful initiation of further infections.

The glycosylation of the influenza strain can disturb its host specificity, virulence, and contagious nature either directly by changing the biological activity of surface proteins (Schulze, 1997), or indirectly by 1) attenuating receptor-binding sites (Gao et al., 2009), 2) masking antigenic sections of the protein (Abe et al., 2004), 3) obstructing the HA protein precursor *via* its cleavage into the disulfide-linked subunits HA1 and HA2 (Ohuchi et al., 1989), and 4) regulating the catalytic activity or preventing proteolytic cleavage of the stalk of NA (Matsuoka et al., 2009). A report revealing the destabilization of the coiled coil of the HA protein due to the buried hydrophilic residue, Thr59, also endorses the sequential and structural level of distortions raised by the mutations threonine-to-serine observed in this study (Lin et al., 2018). Hence, the examined mutation-mediated structural diversification of the HA protein gains importance.

The ESPS characterized for the Indian isolate reported in 2018 revealed significant changes in the electrostatic surface, which is also presumed to render strong binding of HA proteins with the host receptors. The specific mutations observed in HA_Ind-2018_ (for example, S91R, S181T, S200P, I312V, K319T, I421M, and E523D) may increase the fitness of the virus in a new environment and host, which may render a reduced efficacy toward the available treatment. The mutation-mediated adaptability and efficacy of HA_Ind_ proteins of the influenza A (H1N1) virus need to be studied critically.

## 5 Conclusion

In essence, our data emphasize the evolutionary relationship of H1N1 strains circulated in India during the post-pandemic period, 2009–2018, with the A/H1N1pdm09 pandemic reference strain. The present study clearly depicts the presence of frequent mutations in HA_Ind_ proteins of the influenza A virus, which drifted significantly from the reference HA_Cal_ strain A/California/04/2009. In addition, the mutational, structural, and functional characterization of the circulated influenza A strains indicates that the regionally reported mutations in all HA_Ind_ proteins may be associated with their adaptability in sustaining locally for efficient human transmissibility. In India, during the last few decades, a recurrent episode of influenza A virus infection has been reported among humans, which proposes several factors, including 1) the reflection of better detection technologies and, finally, 2) the need for constant surveillance to monitor any changes in the genomic content of the influenza A viruses that could initiate a potential transmission and pronounced virulence among humans. The findings presented here offer a better insight into the development of distinct next-generation therapeutic inhibitors by accounting all observed mutations in the reported isolates.

In this study, the observed mutational drift results in the 1) alteration of receptor-binding domains, 2) generation of new-variant N-glycosylation and epitope-binding sites, and 3) even modifications at the structural level. Molecular investigations, however, are warranted to confirm the binding and antigenic potential of such residue changes at this point and their associated impact on morbidity and mortality as well. Hence, continued surveillance at a national level is required for the early detection of such genetic changes in viruses and the associated secondary emergence of antiviral resistance. Overall, the present work highlights additional information required for the design of more specific inhibitors with increased selectivity against Indian influenza A (H1N1) viruses.

## Data Availability

The datasets presented in this study can be found in online repositories. The names of the repository/repositories and accession number(s) can be found in the article/Supplementary Material.
