# Supplementary material for: Evolution of Indian Influenza A (H1N1) Hemagglutinin Strains: A Comparative Analysis of the Pandemic Californian HA Strain
Source: Front Mol Biosci. 2023 Mar 16;10:1111869. doi: 10.3389/fmolb.2023.1111869 (PMC10061220; doi:10.3389/fmolb.2023.1111869)
Supplement: Supplementary file 3 [file DataSheet1.zip › Supplementary_file/Supplemantary File SF-2.docx]

**Supplementary Data**

**Evolution of Indian Influenza A (H1N1) Hemagglutinin Strains: a comparative analysis of the pandemic Californian HA**

***Shilpa Sri Pushan^1^, Mahesh Samantaray^1^, Muthukumaran Rajagopalan ^2^ and Ramaswami Amutha^1^****

^1^Department of Bioinformatics, Pondicherry University, R. V. Nagar, Kalapet, Puducherry- 605014, India

^2^Department of Biological Sciences and Bioengineering, Indian Institute of Technology Kanpur, Kanpur

* Corresponding author email ID: amutha_ramu@yahoo.com / ramutha@bicpu.edu.in

**Supplementary Table:** Sequence similarity index of HA_Ind_ surface protein of Influenza A (H1N1) virus circulated during 2009 – 2018.

CLUSTALW multiple alignment

(**https://npsa-prabi.ibcp.fr/cgi-bin/align_clustalw.pl)**

**CLUSTALW options used:**
endgaps=1
gapext=0.2
gapopen=10.0
hgapresidues=GPSNDQERK
matrix=gonnet
maxdiv=30
outorder=aligned
pwgapext=0.1
pwgapopen=10.0
pwmatrix=gonnet
type=PROTEIN

**2009_ Alignment data:**Alignment length: 566
Identity (*) : 362 is 63.96 %
Strongly similar (:) : 86 is 15.19 %
Weakly similar (.) : 42 is 7.42 %
Different : 76 is 13.43 %

TOTAL = 86.57 %

**2010_ Alignment data:**
Alignment length: 566
Identity (*) : 509 is 89.93 %
Strongly similar (:) : 31 is 5.48 %
Weakly similar (.) : 8 is 1.41 %
Different : 18 is 3.18 %

TOTAL= 96.82 %

**2011_Alignment data:**
Alignment length: 566
Identity (*) : 537 is 94.88 %
Strongly similar (:) : 16 is 2.83 %
Weakly similar (.) : 5 is 0.88 %
Different : 8 is 1.41 %

TOTAL= 98.59 %

**2012_Alignment data:**
Alignment length: 566
Identity (*) : 500 is 88.34 %
Strongly similar (:) : 35 is 6.18 %
Weakly similar (.) : 17 is 3.00 %
Different : 14 is 2.47 %

TOTAL= 97.53 %

**2013_Alignment data:**
Alignment length: 566
Identity (*) : 529 is 93.46 %
Strongly similar (:) : 22 is 3.89 %
Weakly similar (.) : 5 is 0.88 %
Different : 10 is 1.77 %

TOTAL= 98.23 %

**2014_Alignment data:**
Alignment length: 566
Identity (*) : 557 is 98.41 %
Strongly similar (:) : 5 is 0.88 %
Weakly similar (.) : 2 is 0.35 %
Different : 2 is 0.35 %

TOTAL= 99.65 %

**2015_Alignment data:**
Alignment length: 566
Identity (*) : 532 is 93.99 %
Strongly similar (:) : 13 is 2.30 %
Weakly similar (.) : 9 is 1.59 %
Different : 12 is 2.12 %

TOTAL= 97.88 %

**2016_Alignment data:**
Alignment length: 566
Identity (*) : 546 is 96.47 %
Strongly similar (:) : 11 is 1.94 %
Weakly similar (.) : 4 is 0.71 %
Different : 5 is 0.88 %

TOTAL= 99.12 %

**2017_Alignment data:**
Alignment length: 566
Identity (*) : 493 is 87.10 %
Strongly similar (:) : 36 is 6.36 %
Weakly similar (.) : 15 is 2.65 %
Different : 22 is 3.89 %

TOTAL= 96.11 %

**2018_Alignment data:**
Alignment length: 566
Identity (*) : 548 is 96.82 %
Strongly similar (:) : 11 is 1.94 %
Weakly similar (.) : 3 is 0.53 %
Different : 4 is 0.71 %

TOTAL= 99.29 %

**Alignment data (2009 – 2014):**

Total sequence: 350

Alignment length: 566
Identity (*): 312 is 55.12 %
Strongly similar (:) : 95 is 16.78 %
Weakly similar (.) : 48 is 8.48 %
Different : 111 is 19.61 %

Cumulative sequence similarities = 80.38 %

**Alignment data (2015 – 2018):**

Total sequence: 162
Alignment length: 566
Identity (*) : 447 is 78.98 %
Strongly similar (:) : 54 is 9.54 %
Weakly similar (.) : 26 is 4.59 %
Different : 39 is 6.89 %

Cumulative sequence similarities = 93.11 %
