# Supplementary material for: Evolution of Indian Influenza A (H1N1) Hemagglutinin Strains: A Comparative Analysis of the Pandemic Californian HA Strain
Source: Front Mol Biosci. 2023 Mar 16;10:1111869. doi: 10.3389/fmolb.2023.1111869 (PMC10061220; doi:10.3389/fmolb.2023.1111869)
Supplement: Supplementary file 3 [file DataSheet1.zip › Supplementary_file/Supplemantary_File-S1_Data Collection_name table_HA_H1N1_2009 - 2018.docx]

**Supplementary Data**

**Evolution of Indian Influenza A (H1N1) Hemagglutinin Strains: a comparative analysis of the pandemic Californian HA**

***Shilpa Sri Pushan^1^, Mahesh Samantaray^1^, Muthukumaran Rajagopalan ^2^ and Ramaswamy Amutha^1^****

^1^Department of Bioinformatics, Pondicherry University, R. V. Nagar, Kalapet, Puducherry- 605014, India

^2^Department of Biological Sciences and Bioengineering, Indian Institute of Technology Kanpur, Kanpur

* Corresponding author email ID: amutha_ramu@yahoo.com / ramutha@bicpu.edu.in

**Supplementary Table 1:** Data collection and their NCBI accession numbers of Indian strains of Influenza A (H1N1) virus surface protein hemagglutinin during 2009 – 2018.

| YEAR | ACCESSION NUMBER | STRAIN NAME |
| --- | --- | --- |
| 2009 | \| AEN79398 \| \| --- \| \| ALX27941 \| \| ALX27940 \| \| AKM14710 \| \| AKM14711 \| \| AKM14712 \| \| AKM14713 \| \| AKM14714 \| \| AKM14715 \| \| AKM14716 \| \| AKM14702 \| \| AKM14703 \| \| AKM14704 \| \| AKM14705 \| \| AKM14706 \| \| AKM14707 \| \| AKM14708 \| \| AKM14709 \| \| AJE62444 \| \| ADG57092 \| \| AEM63482 \| \| AJE62451 \| \| AEM63498 \| \| AEM63499 \| \| AEM63506 \| \| ADG57090 \| \| AJE62457 \| \| AJE62458 \| \| AJE62459 \| \| AEM63469 \| \| AEM63470 \| \| AEM63471 \| \| AEM63472 \| \| ADG57087 \| \| ADG57093 \| \| AJE62447 \| \| AJE62449 \| \| AJE62456 \| \| AKS48049 \| \| AIU46626 \| \| AHB72860 \| \| ADG57094 \| \| AJE62454 \| \| AJE62442 \| \| AJE62443 \| \| AII31187 \| \| AII31188 \| \| AII31189 \| \| AEM63475 \| \| AEM63476 \| \| AJE62452 \| \| AEM63478 \| \| AEM63479 \| \| ADG57088 \| \| AEM63505 \| \| ADG57096 \| \| ADG57091 \| \| AIJ10869 \| \| AIJ10870 \| \| AIJ10875 \| \| AIJ10876 \| \| AIJ10871 \| \| AIJ10877 \| \| AIJ10878 \| \| AIJ10872 \| \| AIJ10885 \| \| AIJ10884 \| \| AIJ10887 \| \| AIJ10888 \| \| AIJ10880 \| \| AIJ10904 \| \| AIJ10883 \| \| AIJ10881 \| \| AIJ10882 \| \| AIJ10889 \| \| AIJ10890 \| \| AIJ10859 \| \| AIJ10873 \| \| AIJ10860 \| \| AIJ10861 \| \| AIJ10862 \| \| AIJ10863 \| \| AIJ10864 \| \| AIJ10879 \| \| AIJ10865 \| \| AIJ10874 \| \| AIJ10903 \| \| AIJ10902 \| \| AIJ10901 \| \| AIJ10866 \| \| AIJ10867 \| \| AIJ10868 \| \| AID48433 \| \| AIJ10900 \| \| AIJ10899 \| \| AIJ10898 \| \| AIJ10897 \| \| AIJ10896 \| \| AIJ10858 \| \| AIJ10894 \| \| AIJ10886 \| \| AEM63477 \| \| AEM63483 \| \| ADG57097 \| \| AEM63509 \| \| AEM63473 \| \| AEM63481 \| \| AEM63484 \| \| AEM63485 \| \| AEM63486 \| \| AEM63487 \| \| AEM63488 \| \| ADG57098 \| \| AEM63489 \| \| AEM63490 \| \| ADG57099 \| \| ADG57100 \| \| AEM63491 \| \| AEM63492 \| \| AEM63493 \| \| AEM63494 \| \| AEM63495 \| \| AEM63496 \| \| AEM63507 \| \| AEM63508 \| \| ADG57089 \| \| AJE62448 \| \| AJE62450 \| \| AEM63480 \| \| AJE62453 \| \| AJE62446 \| \| AJE62455 \| \| AJE62445 \| \| ADG57095 \| | \| Influenza A virus (A/Assam/2220/2009(H1N1)) \| \| --- \| \| Influenza A virus (A/Assam/2257/2009(H1N1)) \| \| Influenza A virus (A/Assam/2264/2009(H1N1)) \| \| Influenza A virus (A/Bangalore/10519-13/2009(H1N1)) \| \| Influenza A virus (A/Bangalore/10695-10/2009(H1N1)) \| \| Influenza A virus (A/Bangalore/10698-02/2009(H1N1)) \| \| Influenza A virus (A/Bangalore/11210-01/2009(H1N1)) \| \| Influenza A virus (A/Bangalore/11406-11/2009(H1N1)) \| \| Influenza A virus (A/Bangalore/11658-15/2009(H1N1)) \| \| Influenza A virus (A/Bangalore/12220-18/2009(H1N1)) \| \| Influenza A virus (A/Bangalore/2960-03/2009(H1N1)) \| \| Influenza A virus (A/Bangalore/6053-14/2009(H1N1)) \| \| Influenza A virus (A/Bangalore/6206-12/2009(H1N1)) \| \| Influenza A virus (A/Bangalore/6974-08/2009(H1N1)) \| \| Influenza A virus (A/Bangalore/8330-09/2009(H1N1)) \| \| Influenza A virus (A/Bangalore/8973-04/2009(H1N1)) \| \| Influenza A virus (A/Bangalore/8975-19/2009(H1N1)) \| \| Influenza A virus (A/Bangalore/9274-06/2009(H1N1)) \| \| Influenza A virus (A/Bihar/003/2009(H1N1)) \| \| Influenza A virus (A/Blore/NIV1189/2009(H1N1)) \| \| Influenza A virus (A/Blore/NIV1196/2009(H1N1)) \| \| Influenza A virus (A/Chattisgarh/010/2009(H1N1)) \| \| Influenza A virus (A/Che/NIV245/2009(H1N1)) \| \| Influenza A virus (A/Che/NIV246/2009(H1N1)) \| \| Influenza A virus (A/Che/NIV551/2009(H1N1)) \| \| Influenza A virus (A/Che/NIV658/2009(H1N1)) \| \| Influenza A virus (A/Delhi/016/2009(H1N1)) \| \| Influenza A virus (A/Delhi/017/2009(H1N1)) \| \| Influenza A virus (A/Delhi/018/2009(H1N1)) \| \| Influenza A virus (A/Delhi/NIV0951830/2009(H1N1)) \| \| Influenza A virus (A/Delhi/NIV0951831/2009(H1N1)) \| \| Influenza A virus (A/Delhi/NIV0951832/2009(H1N1)) \| \| Influenza A virus (A/Delhi/NIV0951833/2009(H1N1)) \| \| Influenza A virus (A/Delhi/NIV57/2009(H1N1)) \| \| Influenza A virus (A/Dhule/NIV9433/2009(H1N1)) \| \| Influenza A virus (A/Goa/006/2009(H1N1)) \| \| Influenza A virus (A/Gujarat/008/2009(H1N1)) \| \| Influenza A virus (A/Haryana/015/2009(H1N1)) \| \| Influenza A virus (A/Indore/10/2009(H1N1)) \| \| Influenza A virus (A/Indore/59/2009(H1N1)) \| \| Influenza A virus (A/Jabalpur/112/2009(H1N1)) \| \| Influenza A virus (A/Jalna/NIV9436/2009(H1N1)) \| \| Influenza A virus (A/Jammu and Kashmir/013/2009(H1N1)) \| \| Influenza A virus (A/Karnataka/001/2009(H1N1)) \| \| Influenza A virus (A/Kerala/002/2009(H1N1)) \| \| Influenza A virus (A/Kerala/RGCBA923/2009(H1N1)) \| \| Influenza A virus (A/Kerala/RGCBB360/2009(H1N1)) \| \| Influenza A virus (A/Kerala/RGCBC291/2009(H1N1)) \| \| Influenza A virus (A/Kol/NIV105774/2009(H1N1)) \| \| Influenza A virus (A/Kol/NIV105777/2009(H1N1)) \| \| Influenza A virus (A/Madhya Pradesh/011/2009(H1N1)) \| \| Influenza A virus (A/Mum/NIV1126/2009(H1N1)) \| \| Influenza A virus (A/Mum/NIV1134/2009(H1N1)) \| \| Influenza A virus (A/Mum/NIV261/2009(H1N1)) \| \| Influenza A virus (A/Mum/NIV398/2009(H1N1)) \| \| Influenza A virus (A/Mum/NIV9312/2009(H1N1)) \| \| Influenza A virus (A/Mum/NIV968/2009(H1N1)) \| \| Influenza A virus (A/Mumbai/151/2009(H1N1)) \| \| Influenza A virus (A/Mumbai/154/2009(H1N1)) \| \| Influenza A virus (A/Mumbai/157/2009(H1N1)) \| \| Influenza A virus (A/Mumbai/162/2009(H1N1)) \| \| Influenza A virus (A/Mumbai/163/2009(H1N1)) \| \| Influenza A virus (A/Mumbai/173/2009(H1N1)) \| \| Influenza A virus (A/Mumbai/178/2009(H1N1)) \| \| Influenza A virus (A/Mumbai/180/2009(H1N1)) \| \| Influenza A virus (A/Mumbai/183/2009(H1N1)) \| \| Influenza A virus (A/Mumbai/193/2009(H1N1)) \| \| Influenza A virus (A/Mumbai/2236/2009(H1N1)) \| \| Influenza A virus (A/Mumbai/2244/2009(H1N1)) \| \| Influenza A virus (A/Mumbai/238/2009(H1N1)) \| \| Influenza A virus (A/Mumbai/2381/2009(H1N1)) \| \| Influenza A virus (A/Mumbai/260/2009(H1N1)) \| \| Influenza A virus (A/Mumbai/262/2009(H1N1)) \| \| Influenza A virus (A/Mumbai/264/2009(H1N1)) \| \| Influenza A virus (A/Mumbai/2867/2009(H1N1)) \| \| Influenza A virus (A/Mumbai/2877/2009(H1N1)) \| \| Influenza A virus (A/Mumbai/3011/2009(H1N1)) \| \| Influenza A virus (A/Mumbai/3026/2009(H1N1)) \| \| Influenza A virus (A/Mumbai/3043/2009(H1N1)) \| \| Influenza A virus (A/Mumbai/3049/2009(H1N1)) \| \| Influenza A virus (A/Mumbai/3051/2009(H1N1)) \| \| Influenza A virus (A/Mumbai/3071/2009(H1N1)) \| \| Influenza A virus (A/Mumbai/3072/2009(H1N1)) \| \| Influenza A virus (A/Mumbai/3086/2009(H1N1)) \| \| Influenza A virus (A/Mumbai/3087/2009(H1N1)) \| \| Influenza A virus (A/Mumbai/3109/2009(H1N1)) \| \| Influenza A virus (A/Mumbai/3133/2009(H1N1)) \| \| Influenza A virus (A/Mumbai/3139/2009(H1N1)) \| \| Influenza A virus (A/Mumbai/3140/2009(H1N1)) \| \| Influenza A virus (A/Mumbai/3158/2009(H1N1)) \| \| Influenza A virus (A/Mumbai/3165/2009(H1N1)) \| \| Influenza A virus (A/Mumbai/3166/2009(H1N1)) \| \| Influenza A virus (A/Mumbai/3212/2009(H1N1)) \| \| Influenza A virus (A/Mumbai/3264/2009(H1N1)) \| \| Influenza A virus (A/Mumbai/3411/2009(H1N1)) \| \| Influenza A virus (A/Mumbai/3413/2009(H1N1)) \| \| Influenza A virus (A/Mumbai/3417/2009(H1N1)) \| \| Influenza A virus (A/Mumbai/4066/2009(H1N1)) \| \| Influenza A virus (A/Mumbai/4109/2009(H1N1)) \| \| Influenza A virus (A/Mumbai/4467/2009(H1N1)) \| \| Influenza A virus (A/Mumbai/925/2009(H1N1)) \| \| Influenza A virus (A/Ngp/NIV11203/2009(H1N1)) \| \| Influenza A virus (A/Ngp/NIV14725/2009(H1N1)) \| \| Influenza A virus (A/Nsk/NIV10348/2009(H1N1)) \| \| Influenza A virus (A/Nsk/NIV9968/2009(H1N1)) \| \| Influenza A virus (A/Pune/NIV0953725/2009(H1N1)) \| \| Influenza A virus (A/Pune/NIV1166/2009(H1N1)) \| \| Influenza A virus (A/Pune/NIV14886/2009(H1N1)) \| \| Influenza A virus (A/Pune/NIV15492/2009(H1N1)) \| \| Influenza A virus (A/Pune/NIV161/2009(H1N1)) \| \| Influenza A virus (A/Pune/NIV19609/2009(H1N1)) \| \| Influenza A virus (A/Pune/NIV19934/2009(H1N1)) \| \| Influenza A virus (A/Pune/NIV20007/2009(H1N1)) \| \| Influenza A virus (A/Pune/NIV20069/2009(H1N1)) \| \| Influenza A virus (A/Pune/NIV21090/2009(H1N1)) \| \| Influenza A virus (A/Pune/NIV21115/2009(H1N1)) \| \| Influenza A virus (A/Pune/NIV21123/2009(H1N1)) \| \| Influenza A virus (A/Pune/NIV21139/2009(H1N1)) \| \| Influenza A virus (A/Pune/NIV21159/2009(H1N1)) \| \| Influenza A virus (A/Pune/NIV21163/2009(H1N1)) \| \| Influenza A virus (A/Pune/NIV21193/2009(H1N1)) \| \| Influenza A virus (A/Pune/NIV21218/2009(H1N1)) \| \| Influenza A virus (A/Pune/NIV21259/2009(H1N1)) \| \| Influenza A virus (A/Pune/NIV652/2009(H1N1)) \| \| Influenza A virus (A/Pune/NIV759/2009(H1N1)) \| \| Influenza A virus (A/Pune/NIV807/2009(H1N1)) \| \| Influenza A virus (A/Punjab/007/2009(H1N1)) \| \| Influenza A virus (A/Rajasthan/009/2009(H1N1)) \| \| Influenza A virus (A/Rtng/NIV11650/2009(H1N1)) \| \| Influenza A virus (A/Tamil Naidu/012/2009(H1N1)) \| \| Influenza A virus (A/Uttar Pradesh/005/2009(H1N1)) \| \| Influenza A virus (A/Uttrakhand/014/2009(H1N1)) \| \| Influenza A virus (A/West Bengal/004/2009(H1N1)) \| \| Influenza A virus (A/Ytml/NIV9438/2009(H1N1)) \| |
| 2010 | \| AJE62461 \| \| --- \| \| AEN79399 \| \| AEM63501 \| \| AKM14717 \| \| AKM14718 \| \| AKM14719 \| \| AKM14720 \| \| AKM14721 \| \| AKM14722 \| \| AKM14723 \| \| AKM14724 \| \| AKM14725 \| \| AKM14726 \| \| AKM14727 \| \| AKM14728 \| \| AKM14729 \| \| AKM14730 \| \| AKM14731 \| \| AKS48052 \| \| AJE62466 \| \| AJE62467 \| \| AJE62477 \| \| AJE62478 \| \| AJE62479 \| \| AJE62480 \| \| AJE62468 \| \| AJE62475 \| \| AJE62476 \| \| AJE62469 \| \| AJE62470 \| \| AEM63502 \| \| ADX31425 \| \| ADX31424 \| \| AHB72861 \| \| AKS48050 \| \| AKS48051 \| \| AIU46628 \| \| AJE62462 \| \| AJE62463 \| \| AJE62464 \| \| AII31190 \| \| AII31191 \| \| AIU46627 \| \| AEM63500 \| \| AJE62465 \| \| AIJ10893 \| \| AIJ10891 \| \| AIJ10892 \| \| AIJ10895 \| \| AEM63504 \| \| AEM63497 \| \| AEM63503 \| \| AJE62471 \| \| AJE62472 \| \| AJE62460 \| \| AJE62473 \| \| AJE62474 \| \| AEM63474 \| | \| Influenza A virus (A/Assam/020/2010(H1N1)) \| \| --- \| \| Influenza A virus (A/Assam/2590/2010(H1N1)) \| \| Influenza A virus (A/Awb/NIV25611/2010(H1N1)) \| \| Influenza A virus (A/Bangalore/3934-28/2010(H1N1)) \| \| Influenza A virus (A/Bangalore/4080-23/2010(H1N1)) \| \| Influenza A virus (A/Bangalore/4161-22/2010(H1N1)) \| \| Influenza A virus (A/Bangalore/4163-30/2010(H1N1)) \| \| Influenza A virus (A/Bangalore/4895-25/2010(H1N1)) \| \| Influenza A virus (A/Bangalore/4976-17/2010(H1N1)) \| \| Influenza A virus (A/Bangalore/4985-07/2010(H1N1)) \| \| Influenza A virus (A/Bangalore/5274-20/2010(H1N1)) \| \| Influenza A virus (A/Bangalore/5279-29/2010(H1N1)) \| \| Influenza A virus (A/Bangalore/5531-27/2010(H1N1)) \| \| Influenza A virus (A/Bangalore/5532-05/2010(H1N1)) \| \| Influenza A virus (A/Bangalore/5674-21/2010(H1N1)) \| \| Influenza A virus (A/Bangalore/5712-24/2010(H1N1)) \| \| Influenza A virus (A/Bangalore/6106-16/2010(H1N1)) \| \| Influenza A virus (A/Bangalore/6846-26/2010(H1N1)) \| \| Influenza A virus (A/Bhopal/1544/2010(H1N1)) \| \| Influenza A virus (A/Chattisgarh/025/2010(H1N1)) \| \| Influenza A virus (A/Chattisgarh/026/2010(H1N1)) \| \| Influenza A virus (A/Delhi/036/2010(H1N1)) \| \| Influenza A virus (A/Delhi/037/2010(H1N1)) \| \| Influenza A virus (A/Delhi/038/2010(H1N1)) \| \| Influenza A virus (A/Delhi/039/2010(H1N1)) \| \| Influenza A virus (A/Goa/027/2010(H1N1)) \| \| Influenza A virus (A/Goa/034/2010(H1N1)) \| \| Influenza A virus (A/Goa/035/2010(H1N1)) \| \| Influenza A virus (A/Haryana/028/2010(H1N1)) \| \| Influenza A virus (A/Haryana/029/2010(H1N1)) \| \| Influenza A virus (A/Hnl/NIV26271/2010(H1N1)) \| \| Influenza A virus (A/India/Blore/2010(H1N1)) \| \| Influenza A virus (A/India/GWL_DSC/2010(H1N1)) \| \| Influenza A virus (A/Indore/1085/2010(H1N1)) \| \| Influenza A virus (A/Indore/379/2010(H1N1)) \| \| Influenza A virus (A/Jabalpur/1413/2010(H1N1)) \| \| Influenza A virus (A/Jabalpur/543/2010(H1N1)) \| \| Influenza Avirus(A/JammuandKashmir/021/2010(H1N1)) \| \| Influenza Avirus(A/JammuandKashmir/022/2010(H1N1)) \| \| Influenza A virus (A/Kerala/023/2010(H1N1)) \| \| Influenza A virus (A/Kerala/RGCBC699/2010(H1N1)) \| \| Influenza A virus (A/Kerala/RGCBC700/2010(H1N1)) \| \| Influenza A virus (A/Khargone/293/2010(H1N1)) \| \| Influenza A virus (A/Lur/NIV24770/2010(H1N1)) \| \| Influenza A virus (A/Madhya Pradesh/024/2010(H1N1)) \| \| Influenza A virus (A/Mumbai/4593/2010(H1N1)) \| \| Influenza A virus (A/Mumbai/4916/2010(H1N1)) \| \| Influenza A virus (A/Mumbai/4923/2010(H1N1)) \| \| Influenza A virus (A/Mumbai/5116/2010(H1N1)) \| \| Influenza A virus (A/Nanded/NIV29214/2010(H1N1)) \| \| Influenza A virus (A/Ngp/NIV22704/2010(H1N1)) \| \| Influenza A virus (A/Pune/NIV26410/2010(H1N1)) \| \| Influenza A virus (A/Punjab/030/2010(H1N1)) \| \| Influenza A virus (A/Punjab/031/2010(H1N1)) \| \| Influenza A virus (A/Rajasthan/019/2010(H1N1)) \| \| Influenza A virus (A/Uttar Pradesh/032/2010(H1N1)) \| \| Influenza A virus (A/Uttrakhand/033/2010(H1N1)) \| \| Influenza A virus (A/Vadu/NIV1043725/2010(H1N1)) \| |
| 2011 | \| AKM14732 \| \| --- \| \| AKM14733 \| \| AKM14734 \| \| AKM14735 \| \| AKM14736 \| \| AIU46629 \| \| AKS48053 \| \| AJE62491 \| \| AJE62492 \| \| AJE62493 \| \| AJE62494 \| \| AJE62495 \| \| AJE62496 \| \| AJE62497 \| \| AJE62487 \| \| AJE62488 \| \| AJE62489 \| \| AJE62490 \| \| AEX63612 \| \| AEX63611 \| \| AGQ88654 \| \| AGQ88643 \| \| AOR17493 \| \| AJE62485 \| \| AJE62486 \| \| AII31192 \| \| AII31193 \| \| AII31194 \| \| AII31195 \| \| AJE62481 \| \| AJE62482 \| \| AJE62483 \| \| AJE62484 \| | \| Influenza A virus (A/Bangalore/1103-51/2011(H1N1)) \| \| --- \| \| Influenza A virus (A/Bangalore/1326-52/2011(H1N1)) \| \| Influenza A virus (A/Bangalore/1808-53/2011(H1N1)) \| \| Influenza A virus (A/Bangalore/1974-55/2011(H1N1)) \| \| Influenza A virus (A/Bangalore/2327-56/2011(H1N1)) \| \| Influenza A virus (A/Bhopal/1613/2011(H1N1)) \| \| Influenza A virus (A/Bhopal/1664/2011(H1N1)) \| \| Influenza A virus (A/Delhi/050/2011(H1N1)) \| \| Influenza A virus (A/Delhi/051/2011(H1N1)) \| \| Influenza A virus (A/Delhi/052/2011(H1N1)) \| \| Influenza A virus (A/Delhi/053/2011(H1N1)) \| \| Influenza A virus (A/Delhi/054/2011(H1N1)) \| \| Influenza A virus (A/Delhi/055/2011(H1N1)) \| \| Influenza A virus (A/Delhi/056/2011(H1N1)) \| \| Influenza A virus (A/Goa/046/2011(H1N1)) \| \| Influenza A virus (A/Goa/047/2011(H1N1)) \| \| Influenza A virus (A/Goa/048/2011(H1N1)) \| \| Influenza A virus (A/Haryana/049/2011(H1N1)) \| \| Influenza A virus (A/India/GWL01/2011(H1N1)) \| \| Influenza A virus (A/India/GWL02/2011(H1N1)) \| \| Influenza A virus (A/India/P1112874/2011(H1N1)) \| \| Influenza A virus (A/India/P1114854/2011(H1N1)) \| \| Influenza A virus (A/India/P121253/2011(H1N1)) \| \| Influenza A virus (A/Jammu and Kashmir/044/2011(H1N1)) \| \| Influenza A virus (A/Jammu and Kashmir/045/2011(H1N1)) \| \| Influenza A virus (A/Kerala/RGCBE658/2011(H1N1)) \| \| Influenza A virus (A/Kerala/RGCBE679/2011(H1N1)) \| \| Influenza A virus (A/Kerala/RGCBE680/2011(H1N1)) \| \| Influenza A virus (A/Kerala/RGCBF2/2011(H1N1)) \| \| Influenza A virus (A/Punjab/040/2011(H1N1)) \| \| Influenza A virus (A/Punjab/041/2011(H1N1)) \| \| Influenza A virus (A/Punjab/042/2011(H1N1)) \| \| Influenza A virus (A/Punjab/043/2011(H1N1)) \| |
| 2012 | \| AKM14739 \| \| --- \| \| AKM14740 \| \| AKM14741 \| \| AKM14742 \| \| AKM14743 \| \| AKM14744 \| \| AKM14745 \| \| AKM14746 \| \| AKM14747 \| \| AKM14748 \| \| AKM14749 \| \| AKM14750 \| \| AKM14751 \| \| AKM14737 \| \| AKM14738 \| \| AIU46630 \| \| AJE62515 \| \| AJE62516 \| \| AJE62498 \| \| AJE62499 \| \| AJE62500 \| \| AJE62501 \| \| AJE62502 \| \| AJE62503 \| \| AJE62504 \| \| AJE62505 \| \| AJE62506 \| \| AJE62507 \| \| AJE62508 \| \| AJE62509 \| \| AJE62510 \| \| AJE62512 \| \| AJE62513 \| \| AJE62514 \| \| AIU46631 \| \| AJE62517 \| \| AJE62518 \| \| AJE62519 \| \| AGL07681 \| \| AOR17532 \| \| AOR17531 \| \| AOR17528 \| \| AOR17529 \| \| AOR17530 \| \| AGQ88632 \| \| AOR17542 \| \| AOR17535 \| \| AOR17540 \| \| AOR17537 \| \| AOR17536 \| \| AOR17533 \| \| AOR17544 \| \| AGQ88621 \| \| AOR17492 \| \| AGQ88610 \| \| AGQ88599 \| \| AGQ88588 \| \| AOR17494 \| \| AGQ88577 \| \| AOR17496 \| \| AOR17508 \| \| AOR17497 \| \| AOR17511 \| \| AOR17499 \| \| AOR17502 \| \| AOR17498 \| \| AOR17510 \| \| AOR17503 \| \| AOR17495 \| \| AOR17488 \| \| AOR17512 \| \| AOR17501 \| \| AOR17487 \| \| AOR17507 \| \| AOR17505 \| \| AOR17489 \| \| AOR17504 \| \| AOR17506 \| \| AOR17509 \| \| AOR17491 \| \| AOR17500 \| \| AOR17490 \| \| AGQ88566 \| \| AOR17541 \| \| AOR17538 \| \| AOR17539 \| \| AGQ88555 \| \| AIU46632 \| \| AIU46633 \| \| AII31196 \| \| AII31197 \| \| AII31198 \| \| AJE62511 \| | \| Influenza A virus (A/Bangalore/1155-33/2012(H1N1)) \| \| --- \| \| Influenza A virus (A/Bangalore/1428-35/2012(H1N1)) \| \| Influenza A virus (A/Bangalore/1491-34/2012(H1N1)) \| \| Influenza A virus (A/Bangalore/1980-42/2012(H1N1)) \| \| Influenza A virus (A/Bangalore/2008-43/2012(H1N1)) \| \| Influenza A virus (A/Bangalore/2071-38/2012(H1N1)) \| \| Influenza A virus (A/Bangalore/2285-44/2012(H1N1)) \| \| Influenza A virus (A/Bangalore/2629-37/2012(H1N1)) \| \| Influenza A virus (A/Bangalore/2813-36/2012(H1N1)) \| \| Influenza A virus (A/Bangalore/3361-46/2012(H1N1)) \| \| Influenza A virus (A/Bangalore/3432-40/2012(H1N1)) \| \| Influenza A virus (A/Bangalore/3666-41/2012(H1N1)) \| \| Influenza A virus (A/Bangalore/3925-45/2012(H1N1)) \| \| Influenza A virus (A/Bangalore/597-39/2012(H1N1)) \| \| Influenza A virus (A/Bangalore/697-32/2012(H1N1)) \| \| Influenza A virus (A/Bhopal/1697/2012(H1N1)) \| \| Influenza A virus (A/Chattisgarh/074/2012(H1N1)) \| \| Influenza A virus (A/Chattisgarh/075/2012(H1N1)) \| \| Influenza A virus (A/Delhi/057/2012(H1N1)) \| \| Influenza A virus (A/Delhi/058/2012(H1N1)) \| \| Influenza A virus (A/Delhi/059/2012(H1N1)) \| \| Influenza A virus (A/Delhi/060/2012(H1N1)) \| \| Influenza A virus (A/Delhi/061/2012(H1N1)) \| \| Influenza A virus (A/Delhi/062/2012(H1N1)) \| \| Influenza A virus (A/Delhi/063/2012(H1N1)) \| \| Influenza A virus (A/Delhi/064/2012(H1N1)) \| \| Influenza A virus (A/Delhi/065/2012(H1N1)) \| \| Influenza A virus (A/Delhi/066/2012(H1N1)) \| \| Influenza A virus (A/Delhi/067/2012(H1N1)) \| \| Influenza A virus (A/Delhi/068/2012(H1N1)) \| \| Influenza A virus (A/Delhi/069/2012(H1N1)) \| \| Influenza A virus (A/Goa/071/2012(H1N1)) \| \| Influenza A virus (A/Goa/072/2012(H1N1)) \| \| Influenza A virus (A/Goa/073/2012(H1N1)) \| \| Influenza A virus (A/Harda/1718/2012(H1N1)) \| \| Influenza A virus (A/Haryana/076/2012(H1N1)) \| \| Influenza A virus (A/Haryana/077/2012(H1N1)) \| \| Influenza A virus (A/Haryana/078/2012(H1N1)) \| \| Influenza A virus (A/India/Gwl-06/2012(H1N1)) \| \| Influenza A virus (A/India/Kerala1210448/2012(H1N1)) \| \| Influenza A virus (A/India/Kerala1210449/2012(H1N1)) \| \| Influenza A virus (A/India/Kerala128941/2012(H1N1)) \| \| Influenza A virus (A/India/Kerala128942/2012(H1N1)) \| \| Influenza A virus (A/India/Kerala128943/2012(H1N1)) \| \| Influenza A virus (A/India/Nsk12388/2012(H1N1)) \| \| Influenza A virus (A/India/P1210194/2012(H1N1)) \| \| Influenza A virus (A/India/P1210472/2012(H1N1)) \| \| Influenza A virus (A/India/P1210573/2012(H1N1)) \| \| Influenza A virus (A/India/P1210772/2012(H1N1)) \| \| Influenza A virus (A/India/P1210993/2012(H1N1)) \| \| Influenza A virus (A/India/P1213259/2012(H1N1)) \| \| Influenza A virus (A/India/P1213777/2012(H1N1)) \| \| Influenza A virus (A/India/P121716/2012(H1N1)) \| \| Influenza A virus (A/India/P121717/2012(H1N1)) \| \| Influenza A virus (A/India/P121717/2012(H1N1)) \| \| Influenza A virus (A/India/P121773/2012(H1N1)) \| \| Influenza A virus (A/India/P121778/2012(H1N1)) \| \| Influenza A virus (A/India/P121788/2012(H1N1)) \| \| Influenza A virus (A/India/P121939/2012(H1N1)) \| \| Influenza A virus (A/India/P122023/2012(H1N1)) \| \| Influenza A virus (A/India/P122045/2012(H1N1)) \| \| Influenza A virus (A/India/P122059/2012(H1N1)) \| \| Influenza A virus (A/India/P122090/2012(H1N1)) \| \| Influenza A virus (A/India/P122246/2012(H1N1)) \| \| Influenza A virus (A/India/P122300/2012(H1N1)) \| \| Influenza A virus (A/India/P122576/2012(H1N1)) \| \| Influenza A virus (A/India/P122639/2012(H1N1)) \| \| Influenza A virus (A/India/P122654/2012(H1N1)) \| \| Influenza A virus (A/India/P122703/2012(H1N1)) \| \| Influenza A virus (A/India/P122707/2012(H1N1)) \| \| Influenza A virus (A/India/P122975/2012(H1N1)) \| \| Influenza A virus (A/India/P123137/2012(H1N1)) \| \| Influenza A virus (A/India/P123148/2012(H1N1)) \| \| Influenza A virus (A/India/P123167/2012(H1N1)) \| \| Influenza A virus (A/India/P123176/2012(H1N1)) \| \| Influenza A virus (A/India/P123181/2012(H1N1)) \| \| Influenza A virus (A/India/P123187/2012(H1N1)) \| \| Influenza A virus (A/India/P123192/2012(H1N1)) \| \| Influenza A virus (A/India/P123246/2012(H1N1)) \| \| Influenza A virus (A/India/P125155/2012(H1N1)) \| \| Influenza A virus (A/India/P125442/2012(H1N1)) \| \| Influenza A virus (A/India/P125709/2012(H1N1)) \| \| Influenza A virus (A/India/P12946/2012(H1N1)) \| \| Influenza A virus (A/India/P129932/2012(H1N1)) \| \| Influenza A virus (A/India/P129953/2012(H1N1)) \| \| Influenza A virus (A/India/P129956/2012(H1N1)) \| \| Influenza A virus (A/India/VD122268/2012(H1N1)) \| \| Influenza A virus (A/Jabalpur/1737/2012(H1N1)) \| \| Influenza A virus (A/Jabalpur/1758/2012(H1N1)) \| \| Influenza A virus (A/Kerala/RGCBF294/2012(H1N1)) \| \| Influenza A virus (A/Kerala/RGCBF302/2012(H1N1)) \| \| Influenza A virus (A/Kerala/RGCBF311/2012(H1N1)) \| \| Influenza A virus (A/Uttrakhand/070/2012(H1N1)) \| |
| 2013 | \| AJE62527 \| \| --- \| \| AJE62528 \| \| AJE62529 \| \| AJE62530 \| \| AJE62520 \| \| AJE62521 \| \| AJE62522 \| \| AKE37409 \| \| AGY42549 \| \| AKE37418 \| \| AKE37419 \| \| AGQ88544 \| \| AGQ88442 \| \| AGQ88533 \| \| AGQ88522 \| \| AKE37436 \| \| AKE37448 \| \| AHB72862 \| \| AIU46634 \| \| AIU46635 \| \| AJE62525 \| \| AJE62526 \| \| AKS48054 \| \| AJE62531 \| \| AJE62532 \| \| AJE62523 \| \| AJE62524 \| | \| Influenza A virus (A/Delhi/086/2013(H1N1)) \| \| --- \| \| Influenza A virus (A/Delhi/087/2013(H1N1)) \| \| Influenza A virus (A/Delhi/088/2013(H1N1)) \| \| Influenza A virus (A/Delhi/089/2013(H1N1)) \| \| Influenza A virus (A/Haryana/079/2013(H1N1)) \| \| Influenza A virus (A/Haryana/080/2013(H1N1)) \| \| Influenza A virus (A/Haryana/081/2013(H1N1)) \| \| Influenza A virus (A/India/Alp135125/2013(H1N1)) \| \| Influenza A virus (A/India/GWL-13/2013(H1N1)) \| \| Influenza A virus (A/India/Nag1320058/2013(H1N1)) \| \| Influenza A virus (A/India/Nag1320061/2013(H1N1)) \| \| Influenza A virus (A/India/Nag132467/2013(H1N1)) \| \| Influenza A virus (A/India/P131027/2013(H1N1)) \| \| Influenza A virus (A/India/P131845/2013(H1N1)) \| \| Influenza A virus (A/India/P132194/2013(H1N1)) \| \| Influenza A virus (A/India/Pun1312095/2013(H1N1)) \| \| Influenza A virus (A/India/Pun1318508/2013(H1N1)) \| \| Influenza A virus (A/Indore/2683/2013(H1N1)) \| \| Influenza A virus (A/Indore/2820/2013(H1N1)) \| \| Influenza A virus (A/Itarsi/2934/2013(H1N1)) \| \| Influenza A virus (A/Jammu and Kashmir/084/2013(H1N1)) \| \| Influenza A virus (A/Jammu and Kashmir/085/2013(H1N1)) \| \| Influenza A virus (A/Ujjain/2558/2013(H1N1)) \| \| Influenza A virus (A/Uttar Pradesh/090/2013(H1N1)) \| \| Influenza A virus (A/Uttar Pradesh/091/2013(H1N1)) \| \| Influenza A virus (A/Uttrakhand/082/2013(H1N1)) \| \| Influenza A virus (A/Uttrakhand/083/2013(H1N1)) \| |
| 2014 | \| AKE37501 \| \| --- \| \| AKE37493 \| \| AKE37494 \| \| ARG42801 \| \| ARG42802 \| | \| Influenza A virus (A/India/Che147504/2014(H1N1)) \| \| --- \| \| Influenza A virus (A/India/Pun14549/2014(H1N1)) \| \| Influenza A virus (A/India/Pun14584/2014(H1N1)) \| \| InfluenzaAvirus(A/Kerala/RGCB140815/2014(H1N1)) \| \| InfluenzaAvirus(A/Kerala/RGCB141437/2014(H1N1)) \| |
| 2015 | \| ALA50342 \| \| --- \| \| ALA50343 \| \| ALD18975 \| \| AKS48057 \| \| ALK80387 \| \| ALK80385 \| \| ALK80389 \| \| ALK80390 \| \| ALK80386 \| \| ALK80388 \| \| AMM43281 \| \| AOO54230 \| \| AOO54231 \| \| AOO54232 \| \| AOO54233 \| \| AOO54234 \| \| AOO54235 \| \| AMM43282 \| \| AMM43283 \| \| AMM43284 \| \| AOO54236 \| \| AOO54237 \| \| AOO54238 \| \| AOO54239 \| \| AOO54180 \| \| AOO54190 \| \| AOO54200 \| \| AOO54210 \| \| AOO54220 \| \| AMM43289 \| \| AMM43290 \| \| AMM43285 \| \| AMM43286 \| \| AMM43287 \| \| AMM43288 \| \| AKE37508 \| \| AKE37509 \| \| AKE37507 \| \| AKE37518 \| \| AKE37510 \| \| AKE37511 \| \| AKE37512 \| \| AKE37514 \| \| AKE37513 \| \| AKE37516 \| \| AKE37515 \| \| AKE37517 \| \| AKS48055 \| \| AKS48056 \| \| ARG42800 \| \| ARG42799 \| \| AMU04342 \| \| ALA50341 \| | \| Influenza A virus (A/Barwani/6024/2015(H1N1)) \| \| --- \| \| Influenza A virus (A/Betul/6515/2015(H1N1)) \| \| Influenza A virus (A/Bhopal/3500/2015(H1N1)) \| \| Influenza A virus (A/Dewas/4497/2015(H1N1)) \| \| Influenza A virus (A/India/DRDE_GWL703/2015(H1N1)) \| \| Influenza A virus (A/India/DRDE_GWL719/2015(H1N1)) \| \| Influenza A virus (A/India/DRDE_GWL721/2015(H1N1)) \| \| Influenza A virus (A/India/DRDE_GWL812/2015(H1N1)) \| \| Influenza A virus (A/India/DRDE_GWL897/2015(H1N1)) \| \| Influenza A virus (A/India/DRDE_GWL989/2015(H1N1)) \| \| Influenza A virus (A/India/Kol-3527/2015(H1N1)) \| \| Influenza A virus (A/India/Kol-3828/2015(H1N1)) \| \| Influenza A virus (A/India/Kol-3846/2015(H1N1)) \| \| Influenza A virus (A/India/Kol-3959/2015(H1N1)) \| \| Influenza A virus (A/India/Kol-4040/2015(H1N1)) \| \| Influenza A virus (A/India/Kol-4122/2015(H1N1)) \| \| Influenza A virus (A/India/Kol-4501/2015(H1N1)) \| \| Influenza A virus (A/India/Kol-4628/2015(H1N1)) \| \| Influenza A virus (A/India/Kol-4632/2015(H1N1)) \| \| Influenza A virus (A/India/Kol-4651/2015(H1N1)) \| \| Influenza A virus (A/India/Kol-4992/2015(H1N1)) \| \| Influenza A virus (A/India/Kol-5018/2015(H1N1)) \| \| Influenza A virus (A/India/Kol-5025/2015(H1N1)) \| \| Influenza A virus (A/India/Kol-5065/2015(H1N1)) \| \| Influenza A virus (A/India/Kol-S4163/2015(H1N1)) \| \| Influenza A virus (A/India/Kol-S4481/2015(H1N1)) \| \| Influenza A virus (A/India/Kol-S4587/2015(H1N1)) \| \| Influenza A virus (A/India/Kol-S4659/2015(H1N1)) \| \| Influenza A virus (A/India/Kol-S4666/2015(H1N1)) \| \| Influenza A virus (A/India/Kol-T15/2015(H1N1)) \| \| Influenza A virus (A/India/Kol-T16/2015(H1N1)) \| \| Influenza A virus (A/India/Kol-T3/2015(H1N1)) \| \| Influenza A virus (A/India/Kol-T4/2015(H1N1)) \| \| Influenza A virus (A/India/Kol-T5/2015(H1N1)) \| \| Influenza A virus (A/India/Kol-T8/2015(H1N1)) \| \| Influenza A virus (A/India/Pun151192/2015(H1N1)) \| \| Influenza A virus (A/India/Pun151214/2015(H1N1)) \| \| Influenza A virus (A/India/Pun151245/2015(H1N1)) \| \| Influenza A virus (A/India/Pun151247/2015(H1N1)) \| \| Influenza A virus (A/India/Pun151268/2015(H1N1)) \| \| Influenza A virus (A/India/Pun151368/2015(H1N1)) \| \| Influenza A virus (A/India/Pun151399/2015(H1N1)) \| \| Influenza A virus (A/India/Pun151508/2015(H1N1)) \| \| Influenza A virus (A/India/Pun153225/2015(H1N1)) \| \| Influenza A virus (A/India/Pun153388/2015(H1N1)) \| \| Influenza A virus (A/India/Pun153389/2015(H1N1)) \| \| Influenza A virus (A/India/Pun153793/2015(H1N1)) \| \| Influenza A virus (A/Indore/3415/2015(H1N1)) \| \| Influenza A virus (A/Indore/3598/2015(H1N1)) \| \| Influenza A virus (A/Kerala/RGCB145385/2015(H1N1)) \| \| Influenza A virus (A/Kerala/RGCB145751/2015(H1N1)) \| \| Influenza A virus (A/Kerala/RGCB145808/2015(H1N1)) \| \| Influenza A virus (A/Ujjain/5448/2015(H1N1)) \| |
| 2016 | \| ASJ82233 \| \| --- \| \| ASJ82234 \| \| ASJ82238 \| \| ASJ82239 \| \| ASJ82240 \| \| ASJ82241 \| \| ASJ82242 \| \| ASJ82243 \| \| ASJ82244 \| \| ASJ82245 \| \| ASJ82246 \| \| ASU06449 \| \| ASU06450 \| \| ASU06451 \| \| ASU06452 \| \| ASU06453 \| \| ASU06454 \| \| ASU06455 \| \| ASU06456 \| \| ASU06457 \| \| ASU06458 \| \| ASU06459 \| | \| Influenza A virus (A/India/C1619574/2016(H1N1)) \| \| --- \| \| Influenza A virus (A/India/C1619573/2016(H1N1)) \| \| Influenza A virus (A/India/P161674/2016(H1N1)) \| \| Influenza A virus (A/India/P167506/2016(H1N1)) \| \| Influenza A virus (A/India/P165002/2016(H1N1)) \| \| Influenza A virus (A/India/P161705/2016(H1N1)) \| \| Influenza A virus (A/India/P161761/2016(H1N1)) \| \| Influenza A virus (A/India/P161819/2016(H1N1)) \| \| Influenza A virus (A/India/P163725/2016(H1N1)) \| \| Influenza A virus (A/India/P164183/2016(H1N1)) \| \| Influenza A virus (A/India/P167512/2016(H1N1)) \| \| Influenza A virus (A/Assam/RMRC_494/2016(H1N1)) \| \| Influenza A virus (A/Assam/RMRC_527/2016(H1N1)) \| \| Influenza A virus (A/Assam/RMRC_598/2016(H1N1)) \| \| Influenza A virus (A/Assam/RMRC_605/2016(H1N1)) \| \| Influenza A virus (A/Assam/RMRC_609/2016(H1N1)) \| \| Influenza A virus (A/Assam/RMRC_709/2016(H1N1)) \| \| Influenza A virus (A/Assam/RMRC_226/2016(H1N1)) \| \| Influenza A virus (A/Assam/RMRC_693/2016(H1N1)) \| \| Influenza A virus (A/Assam/RMRC_711/2016(H1N1)) \| \| Influenza A virus (A/Assam/RMRC_534/2016(H1N1)) \| \| Influenza A virus (A/Assam/RMRC_449/2016(H1N1)) \| |
| 2017 | \| \| ASJ82210 \| \| --- \| \| ASJ82211 \| \| ASJ82212 \| \| ASJ82213 \| \| ASJ82214 \| \| ASJ82215 \| \| ASJ82216 \| \| ASJ82217 \| \| ASJ82218 \| \| ASJ82219 \| \| ASJ82220 \| \| ASJ82221 \| \| ASJ82222 \| \| ASJ82223 \| \| ASJ82224 \| \| ASJ82225 \| \| ASJ82226 \| \| ASJ82227 \| \| ASJ82228 \| \| ASJ82229 \| \| ASJ82230 \| \| ASJ82231 \| \| ASJ82232 \| \| ASJ82235 \| \| ASJ82236 \| \| ASJ82237 \| \| ASR91923 \| \| ASR91924 \| \| ASR91925 \| \| ASR91926 \| \| ASR91927 \| \| ASR91928 \| \| ASR91929 \| \| ATW75053 \| \| ATW75054 \| \| ATW75055 \| \| ATW75056 \| \| ATW75057 \| \| ATW75058 \| \| ATW75059 \| \| ATW75060 \| \| ATW75061 \| \| ATW75062 \| \| ATW75063 \| \| ATW75064 \| \| ATW75065 \| \| ATW75066 \| \| ATW75067 \| \| ATW75068 \| \| ATW75069 \| \| ATW75070 \| \| ATW75071 \| \| ATW75072 \| \| ATW75073 \| \| ATW75074 \| \| ATW75075 \| \| ATW75076 \| \| ATW75077 \| \| ATW75078 \| \| ATW75079 \| \| ATW75080 \| \| ATW75081 \| \| ATW75082 \| \| AUZ20762 \| \| AUZ20763 \| \| AUZ20764 \| \| AUZ20766 \| \| AUZ20767 \| \| AUZ20768 \| \| AUZ20769 \| \| AUZ20770 \| \| AUZ20771 \| \| \| AVZ61244 \| \| --- \| \| AVZ61245 \| \| \|  \| \| \| --- \| --- \| --- \| --- \| --- \| --- \| --- \| --- \| --- \| --- \| --- \| --- \| --- \| --- \| --- \| --- \| --- \| --- \| --- \| --- \| --- \| --- \| --- \| --- \| --- \| --- \| --- \| --- \| --- \| --- \| --- \| --- \| --- \| --- \| --- \| --- \| --- \| --- \| --- \| --- \| --- \| --- \| --- \| --- \| --- \| --- \| --- \| --- \| --- \| --- \| --- \| --- \| --- \| --- \| --- \| --- \| --- \| --- \| --- \| --- \| --- \| --- \| --- \| --- \| --- \| --- \| --- \| --- \| --- \| --- \| --- \| --- \| --- \| --- \| --- \| --- \| --- \| | \| \| Influenza A virus A/India/C1722639/2017(H1N1)) \| \| --- \| \| Influenza A virus A /India/C1722640/2017(H1N1)) \| \| Influenza A virus A/India/Nad1721957/20(H1N1)) \| \| Influenza A virus A/India/C1721710/2017(H1N1)) \| \| Influenza A virus A/India/C1722045/2017(H1N1)) \| \| Influenza A virus A/India/C1721216/2017(H1N1)) \| \| Influenza A virus A/India/C1721030/2017(H1N1)) \| \| Influenza A virus A/India/C1720871/2017(H1N1)) \| \| Influenza A virus A/India/P1720845/2017(H1N1)) \| \| Influenza A virus A/India/C1720872/2017(H1N1)) \| \| Influenza A virus A/India/P1720775/2017(H1N1)) \| \| Influenza A virus A/India/C1721657/2017(H1N1)) \| \| Influenza A virus A/India/C1721552/2017(H1N1)) \| \| Influenza A virus A/India/C1721549/2017(H1N1)) \| \| Influenza A virus A/India/Raj1725717/20(H1N1)) \| \| Influenza A virus A/India/Raj1725726/20(H1N1)) \| \| Influenza A virus A/India/Raj1725720/20(H1N1)) \| \| Influenza A virus A/India/Ahm1725712/20(H1N1)) \| \| Influenza A virus A/India/Ahm1725705/20(H1N1)) \| \| Influenza A virus A/India/Ahm1725707/20(H1N1)) \| \| Influenza A virus A/India/Ahm1725706/20(H1N1)) \| \| Influenza A virus A/India/Hyd1725804/20(H1N1)) \| \| Influenza A virus A/India/Hyd1725803/20(H1N1)) \| \| Influenza A virus A/India/P1722376/2017(H1N1)) \| \| Influenza A virus A/India/P1722256/2017(H1N1)) \| \| Influenza A virus A/India/P1722287/2017(H1N1)) \| \| Influenza A virus A/Kerala/RGCB172158/2(H1N1)) \| \| Influenza A virus A/Kerala/RGCB172171/2(H1N1)) \| \| Influenza A virus A/Kerala/RGCB172326/2(H1N1)) \| \| Influenza A virus A/Kerala/RGCB172108/2(H1N1)) \| \| Influenza A virus A/Kerala/RGCB172247/2(H1N1)) \| \| Influenza A virus A/Kerala/RGCB172263/2(H1N1)) \| \| Influenza A virus A/Kerala/RGCB172302/2(H1N1)) \| \| Influenza A virus A/India/Alap1729368/2(H1N1)) \| \| Influenza A virus A/India/Alap1729371/2(H1N1)) \| \| Influenza A virus A/India/Alap1729373/2(H1N1)) \| \| Influenza A virus A/India/Alap1729379/2(H1N1)) \| \| Influenza A virus A/India/Alap1729375/2(H1N1)) \| \| Influenza A virus A/India/K1730217/2017(H1N1)) \| \| Influenza A virus A/India/K1730225/2017(H1N1)) \| \| Influenza A virus A/India/P1726441/2017(H1N1)) \| \| Influenza A virus A/India/J1726918/2017(H1N1)) \| \| Influenza A virus A/India/J1726926/2017(H1N1)) \| \| Influenza A virus A/India/J1726924/2017(H1N1)) \| \| Influenza A virus A/India/J1726921/2017(H1N1)) \| \| Influenza A virus A/India/J1726923/2017(H1N1)) \| \| Influenza A virus A/India/SOL1726612/20(H1N1)) \| \| Influenza A virus A/India/SOL1726613/20(H1N1)) \| \| Influenza A virus A/India/SOL1726617/20(H1N1)) \| \| Influenza A virus A/India/P1726361/2017(H1N1)) \| \| Influenza A virus A/India/NAN1726753/20(H1N1)) \| \| Influenza A virus A/India/P1726853/2017(H1N1)) \| \| Influenza A virus A/India/P1727283/2017(H1N1)) \| \| Influenza A virus A/India/P1728161/2017(H1N1)) \| \| Influenza A virus A/India/P1728697/2017(H1N1)) \| \| Influenza A virus A/India/P1729358/2017(H1N1)) \| \| Influenza A virus A/India/P1730402/2017(H1N1)) \| \| Influenza A virus A/India/AUR1727078/20(H1N1)) \| \| Influenza A virus A/India/BUL1727942/20(H1N1)) \| \| Influenza A virus A/India/P1727844/2017(H1N1)) \| \| Influenza A virus A/India/S1728630/2017(H1N1)) \| \| Influenza A virus A/India/S1728628/2017(H1N1)) \| \| Influenza A virus A/India/S1730298/2017(H1N1)) \| \| Influenza A virus A/Kolkata/NICED-0001/(H1N1)) \| \| Influenza A virus A/Kolkata/NICED-0002/(H1N1)) \| \| Influenza A virus A/Kolkata/NICED-0003/(H1N1)) \| \| Influenza A virus A/Kolkata/NICED-0005/(H1N1)) \| \| Influenza A virus A/Kolkata/NICED-0006/(H1N1)) \| \| Influenza A virus A/Kolkata/NICED-0007/(H1N1)) \| \| Influenza A virus A/Kolkata/NICED-0008/(H1N1)) \| \| Influenza A virus A/Kolkata/NICED-0009/(H1N1)) \| \| Influenza A virus A/Kolkata/NICED-0010/(H1N1)) \| \| Influenza A virus A/India/1842538/2017(H1N1)) \| \| \| Influenza A virus A/India/1842567/2017(H1N1)) \| \| --- \| \|  \| \| \|  \| \| \| --- \| --- \| --- \| --- \| --- \| --- \| --- \| --- \| --- \| --- \| --- \| --- \| --- \| --- \| --- \| --- \| --- \| --- \| --- \| --- \| --- \| --- \| --- \| --- \| --- \| --- \| --- \| --- \| --- \| --- \| --- \| --- \| --- \| --- \| --- \| --- \| --- \| --- \| --- \| --- \| --- \| --- \| --- \| --- \| --- \| --- \| --- \| --- \| --- \| --- \| --- \| --- \| --- \| --- \| --- \| --- \| --- \| --- \| --- \| --- \| --- \| --- \| --- \| --- \| --- \| --- \| --- \| --- \| --- \| --- \| --- \| --- \| --- \| --- \| --- \| --- \| --- \| --- \| |
| 2018 | \| QEU44874 \| \| --- \| \| QEU44876 \| \| QEU44877A  QEU44883  QEU44880  QEU44882  QEU44879  QEU44878 \| \| QEU44873 \| \| QCP70893  QCP70894  QCP70895 \| \| QCP70896 \| | \| QEU44874 A/India/7044/2018 2018(H1N1)) \| \| --- \| \| QEU44876 A/India/7214/2018 2018(H1N1)) \| \| QEU44877 A/India/7251/2018 2018 (H1N1))  QEU44883 A/India/7339/2018 2018 (H1N1))  QEU44880 A/India/7341/2018 2018 (H1N1))  QEU44882 A/India/7346/2018 2018 (H1N1))  QEU44879 A/India/7347/2018 2018 (H1N1))  QEU44878 A/India/7359/2018 2018(H1N1)) \| \| QEU44873 A/India/7367/2018 2018(H1N1)) \| \| QCP70893 A/India/Che-1851806/2018(H1N1))  QCP70894 A/India/Che-1851809/2018(H1N1))  QCP70895 A/India/Che-1851810/2018(H1N1)) \| \| QCP70896 A/India/Che-1851811/2018(H1N1)) \| |
