# Supplementary material for: Evolution of Indian Influenza A (H1N1) Hemagglutinin Strains: A Comparative Analysis of the Pandemic Californian HA Strain
Source: Front Mol Biosci. 2023 Mar 16;10:1111869. doi: 10.3389/fmolb.2023.1111869 (PMC10061220; doi:10.3389/fmolb.2023.1111869)
Supplement: Supplementary file 3 [file DataSheet1.zip › Supplementary_file/Supplementary File S5.docx]

**Supplementary Figures**

**Evolution of Indian Influenza A (H1N1) Hemagglutinin Strains: a comparative analysis of the pandemic Californian HA**

***Shilpa Sri Pushan^1^, Mahesh Samantaray^1^, Muthukumaran Rajagopalan ^2^ and Ramaswamy Amutha^1^****

^1^Department of Bioinformatics, Pondicherry University, R. V. Nagar, Kalapet, Puducherry- 605014, India

^2^Department of Biological Sciences and Bioengineering, Indian Institute of Technology Kanpur, Kanpur

* Corresponding author email ID: amutha_ramu@yahoo.com / ramutha@bicpu.edu.in


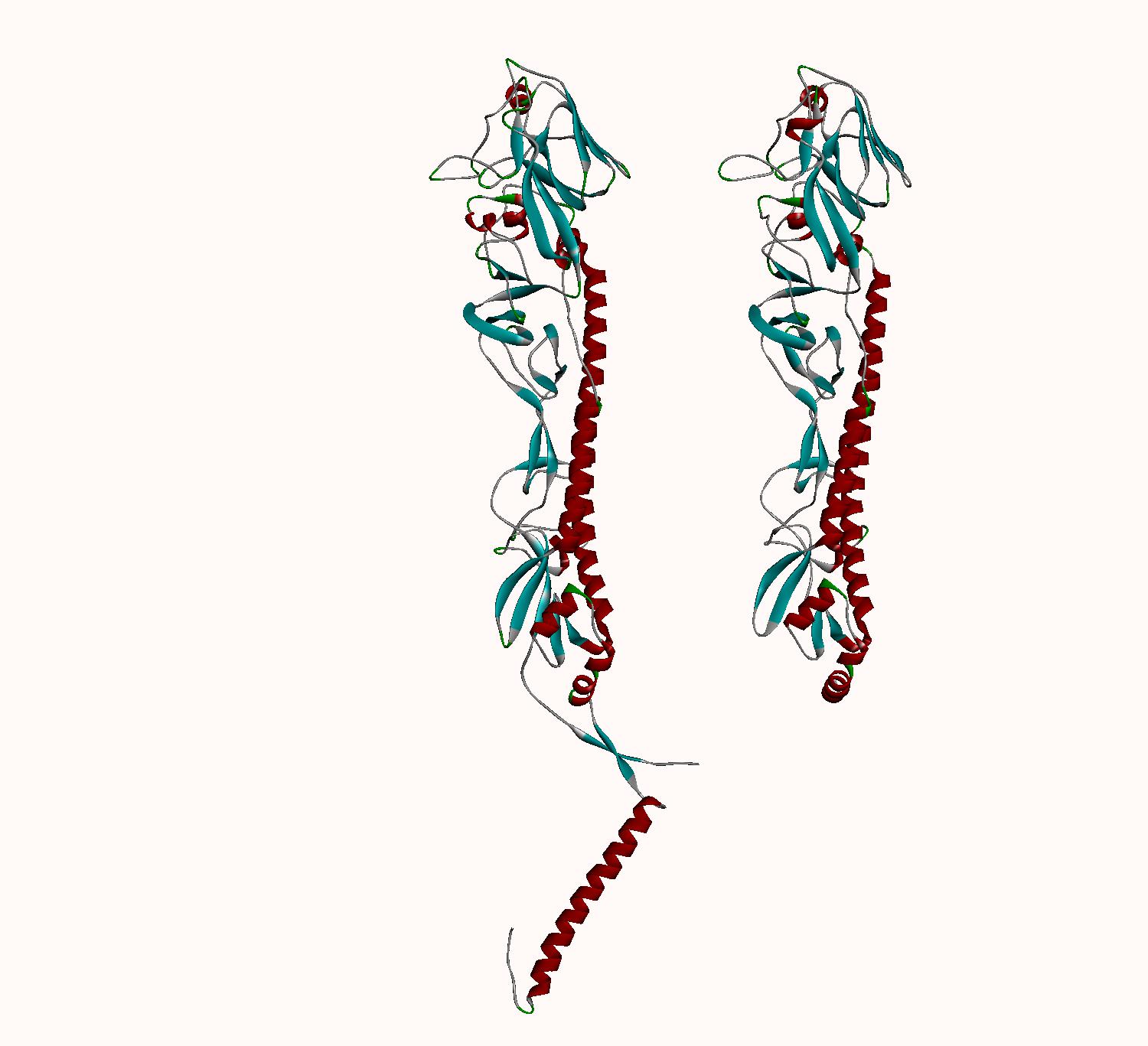

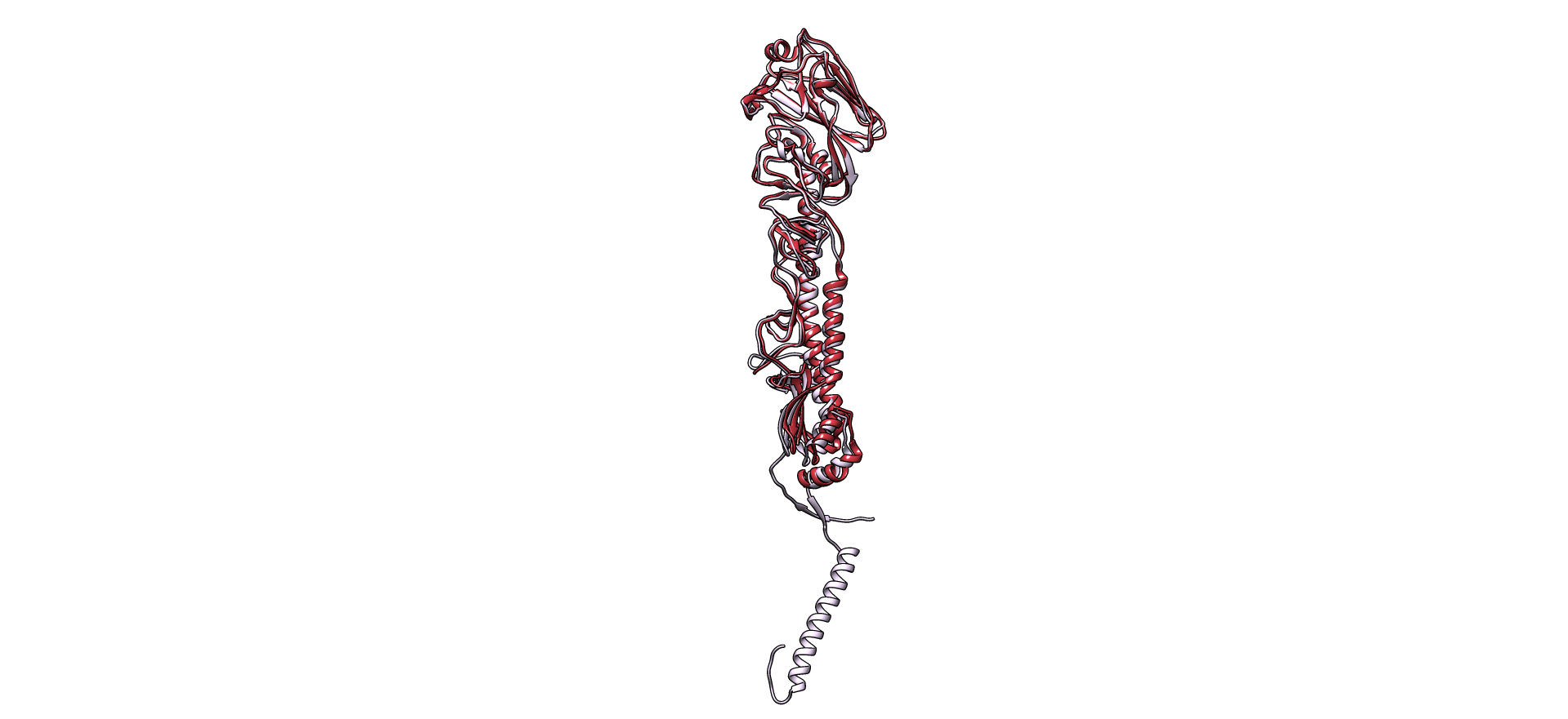


**A**

**B**

**C**

**FIGURE S5.1|** The 3D structure of HA_Cal_ protein of Influenza A (H1N1) virus. Both modelled complete structure (sub set **A**) having 566 amino acids and (ii) crystal structure (sub set **B**) having 506 amino acids (PDB ID: 3LZG) are superimposed as shown in sub set **C**.

**
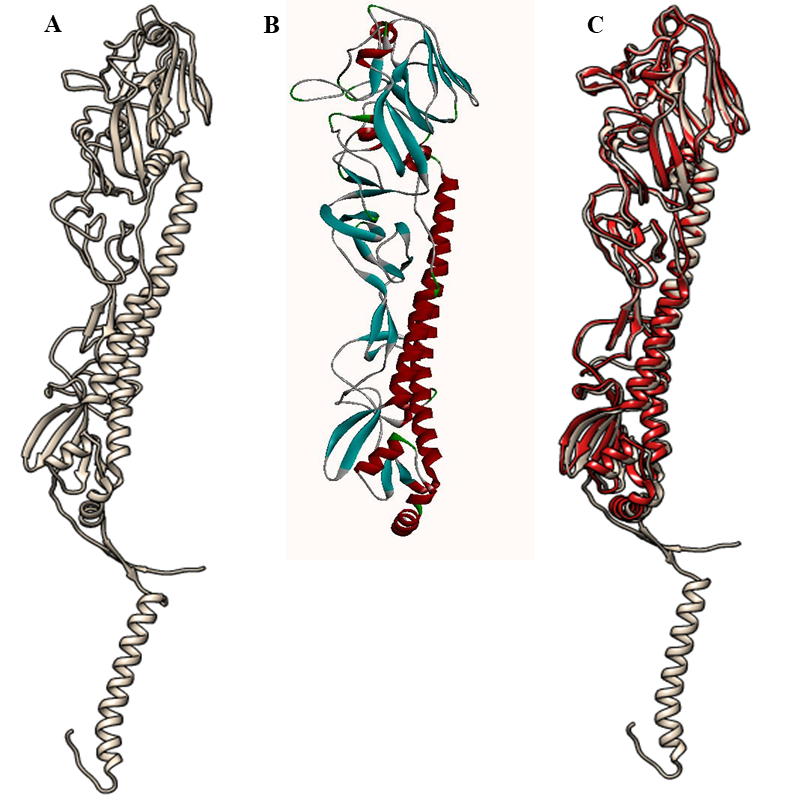
**

**FIGURE S5.2|** The 3D structure of HA_Ind-2018_ protein of Influenza A (H1N1) virus. Both modelled complete structure (sub set **A**) having 566 amino acids and (ii) crystal structure (sub set **B**) having 506 amino acids (PDB ID: 3LZG) are superimposed as shown in sub set **C**.
