# Supplementary material for: Evolution of Indian Influenza A (H1N1) Hemagglutinin Strains: A Comparative Analysis of the Pandemic Californian HA Strain
Source: Front Mol Biosci. 2023 Mar 16;10:1111869. doi: 10.3389/fmolb.2023.1111869 (PMC10061220; doi:10.3389/fmolb.2023.1111869)
Supplement: Supplementary file 6 [file Table1.DOC]

**Supplementary Data**

**Evolution of Indian Influenza A (H1N1) Hemagglutinin Strains: a comparative of against the pandemic Californian HA**

***Shilpa Sri Pushan^1^, Mahesh Samantaray^1^, Muthukumaran Rajagopalan ^2^ and Ramaswamy Amutha^1^****

^1^Department of Bioinformatics, Pondicherry University, R. V. Nagar, Kalapet, Puducherry- 605014, India

^2^Department of Biological Sciences and Bioengineering, Indian Institute of Technology Kanpur, Kanpur

* Corresponding author email ID: [amutha_ramu@yahoo.com](mailto:amutha_ramu@yahoo.com) ramutha@bicpu.edu.in


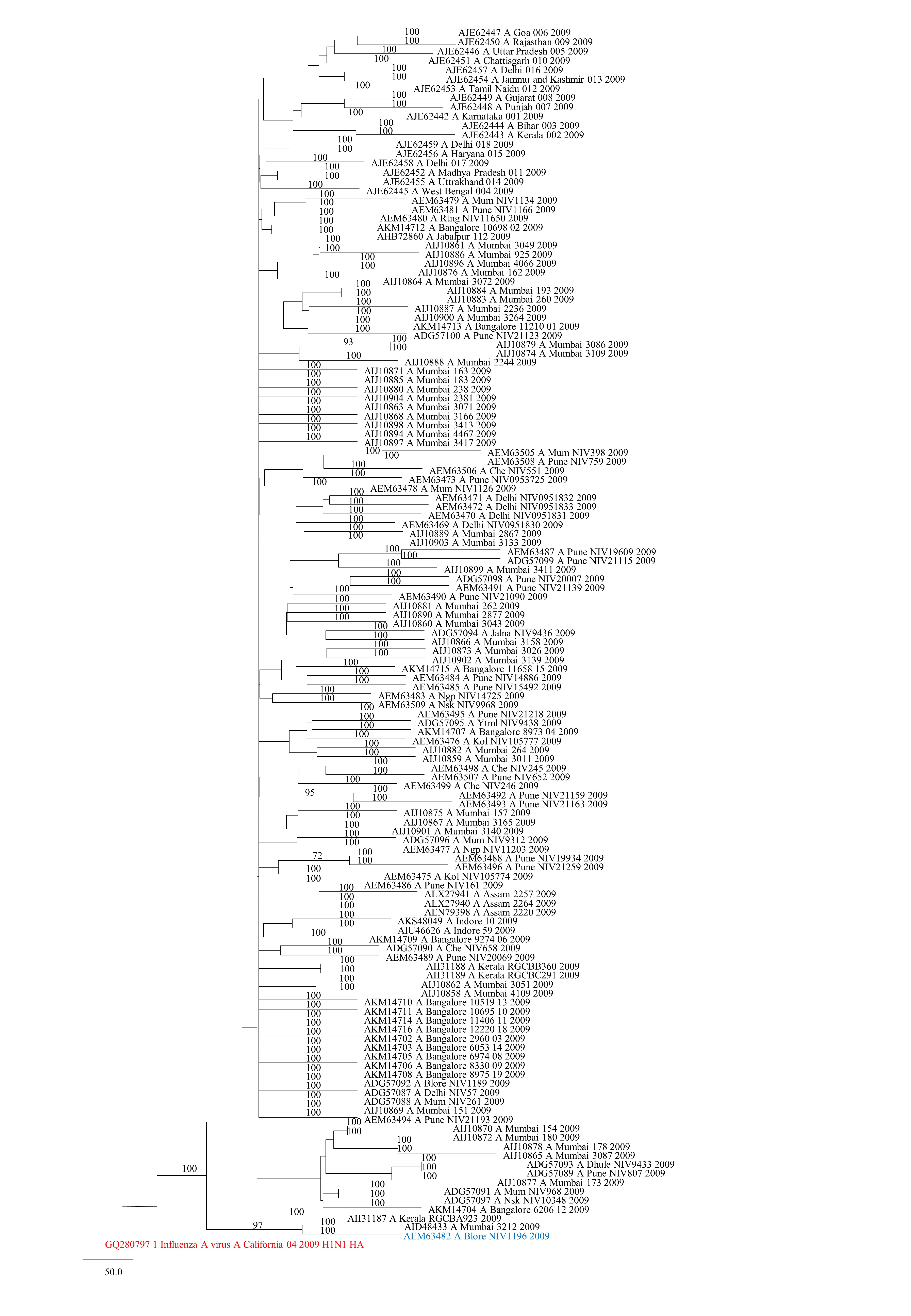


**Supplementary figure 1:** A phylogenetic tree depicting the relationship between the 2009 Indian H1N1-HA strains and pandemic reference strain A/California/04/2009 is given. Bootstrap values greater than 70% have been given above each node for the maximum parsimony method.


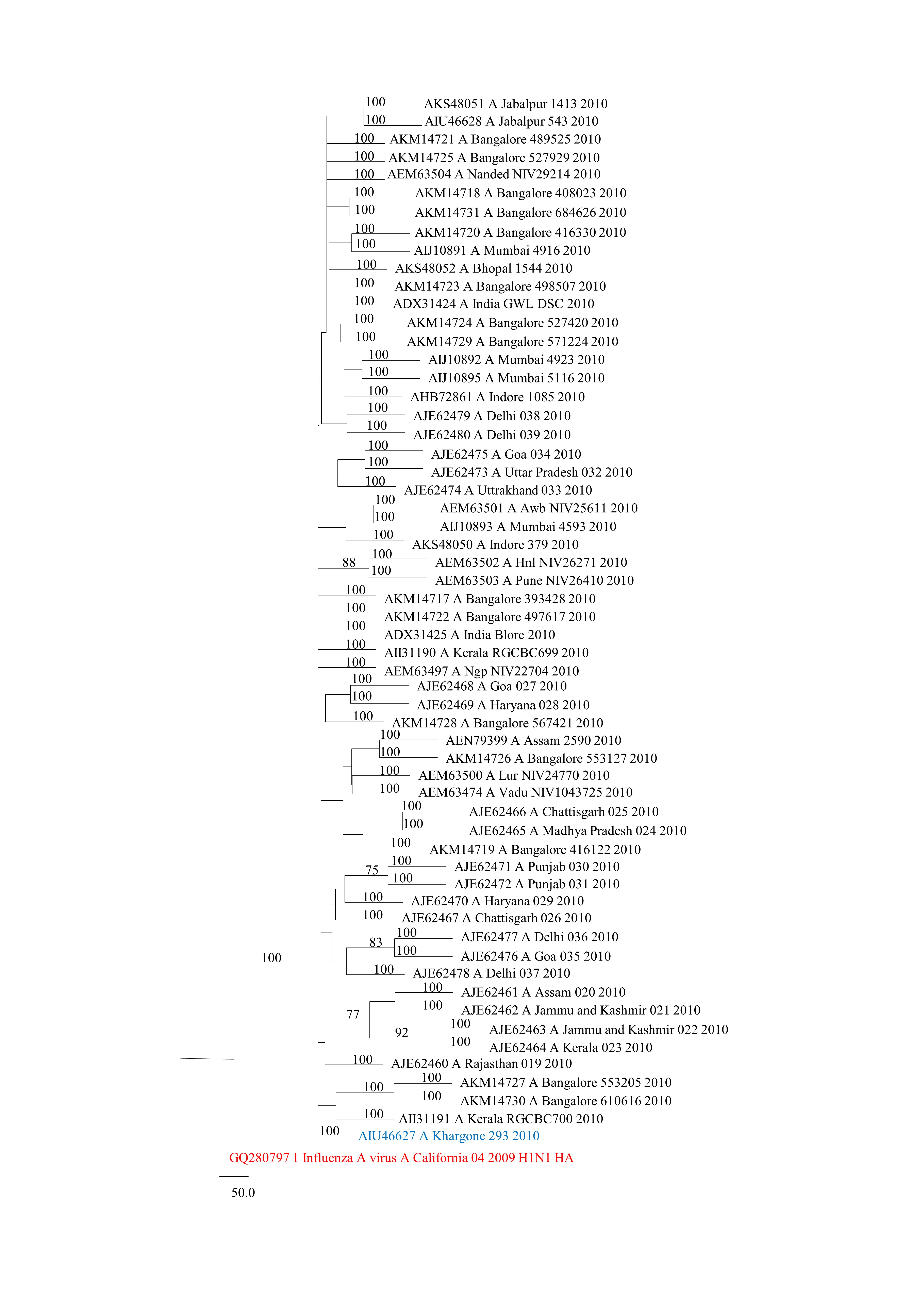


**Supplementary figure 1 (continued):** A phylogenetic tree depicting the relationship between the 2010 Indian H1N1-HA strains and pandemic reference strain A/California/04/2009 is given. Bootstrap values greater than 70% have been given above each node for the maximum parsimony method.


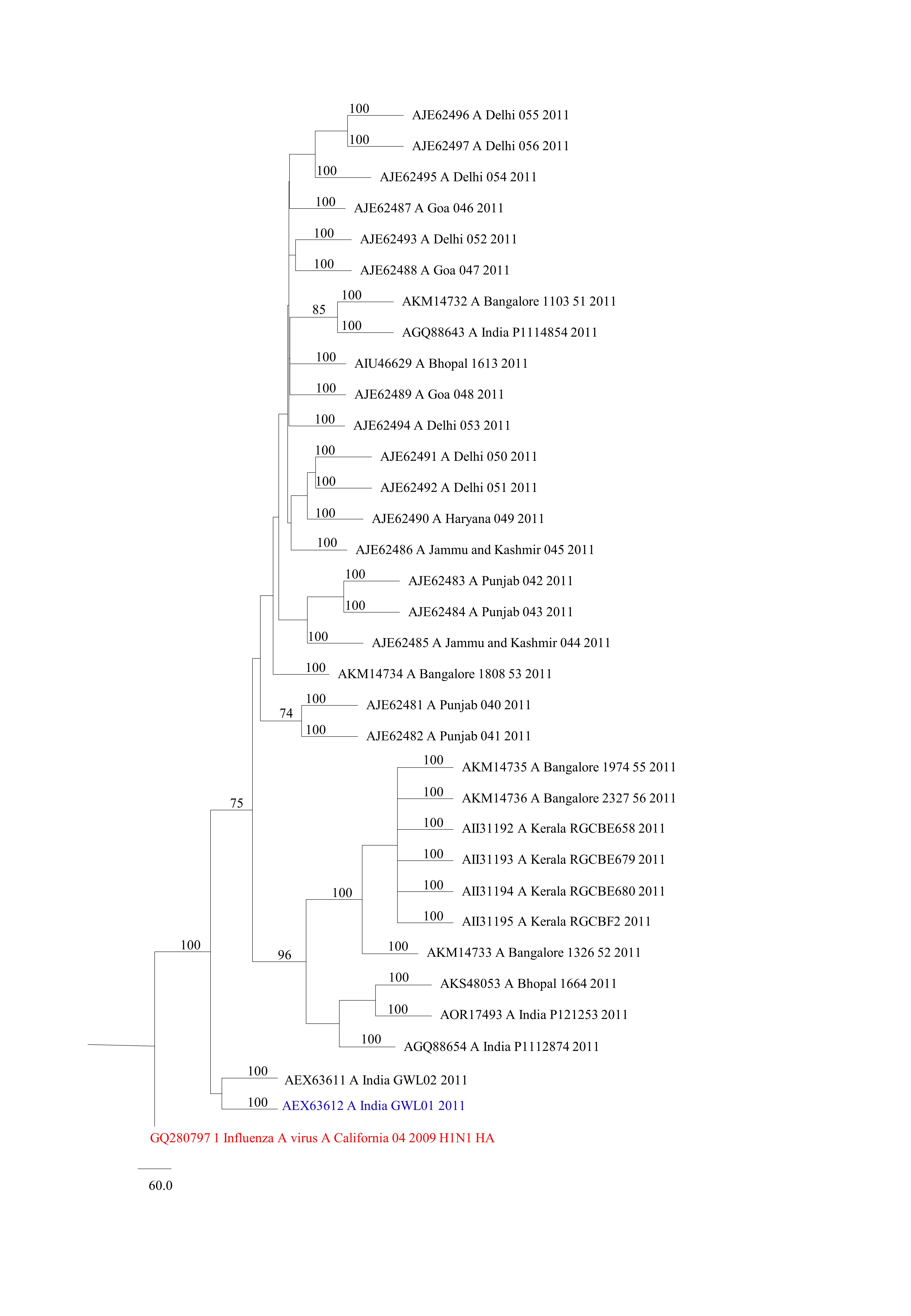


**Supplementary figure 1 (continued):** A phylogenetic tree depicting the relationship between the 2011 Indian H1N1-HA strains and pandemic reference strain A/California/04/2009 is given. Bootstrap values greater than 70% have been given above each node for the maximum parsimony method.


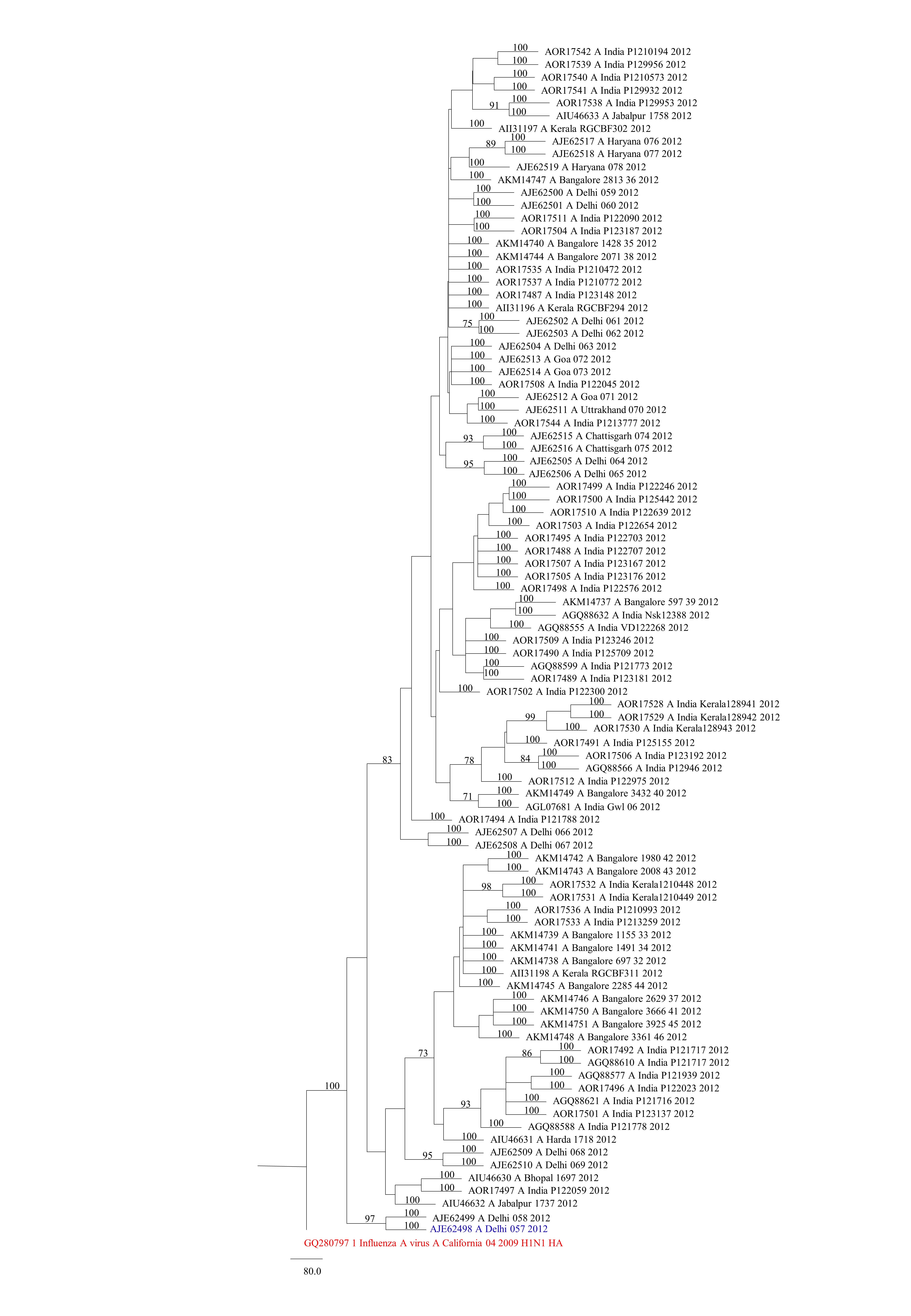


**Supplementary figure 1 (continued):** A phylogenetic tree depicting the relationship between the 2012 Indian H1N1-HA strains and pandemic reference strain A/California/04/2009 is given. Bootstrap values greater than 70% have been given above each node for the maximum parsimony method.


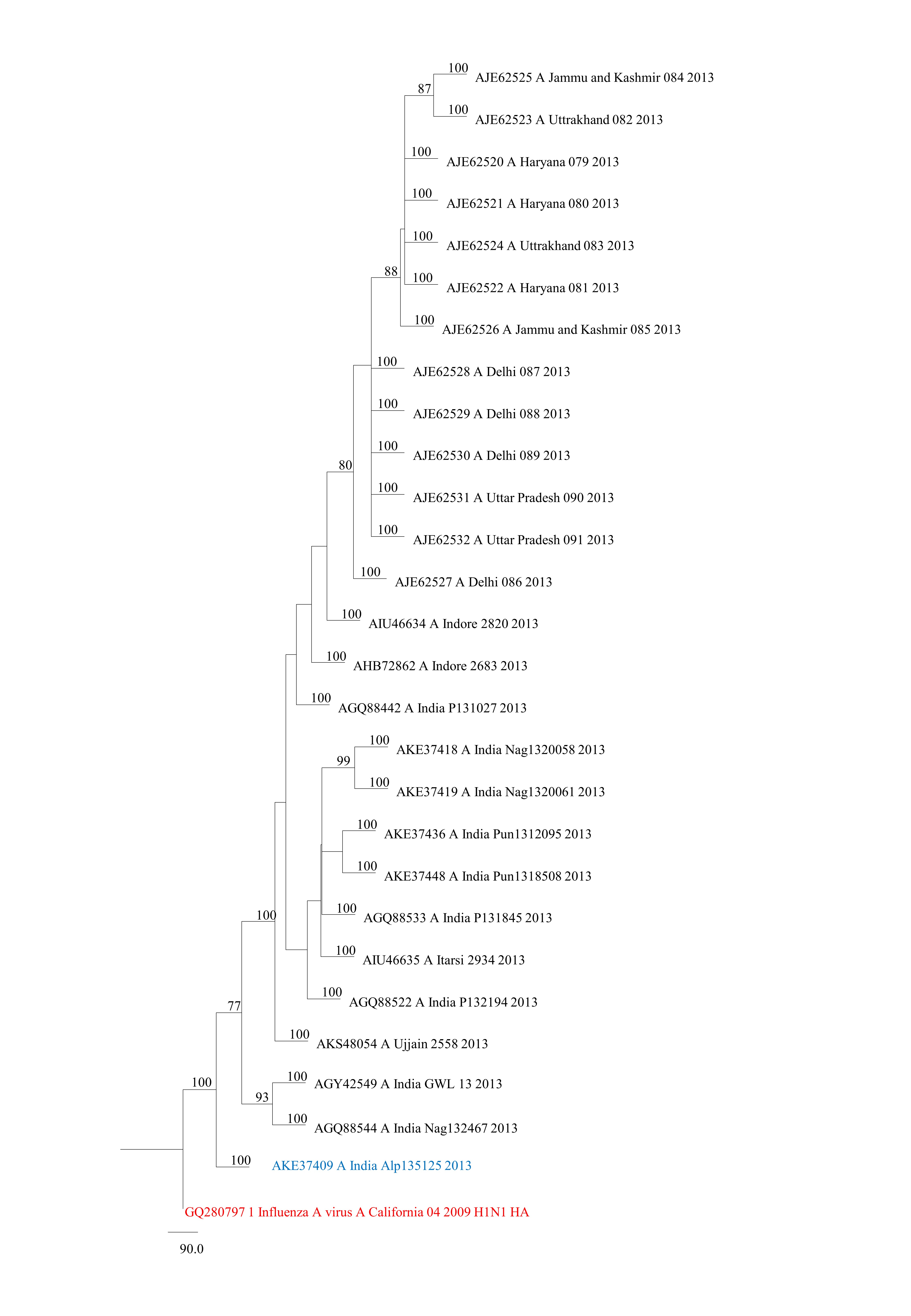


**Supplementary figure 1 (continued):** A phylogenetic tree depicting the relationship between the 2013 Indian H1N1-HA strains and pandemic reference strain A/California/04/2009 is given. Bootstrap values greater than 70% have been given above each node for the maximum parsimony method.


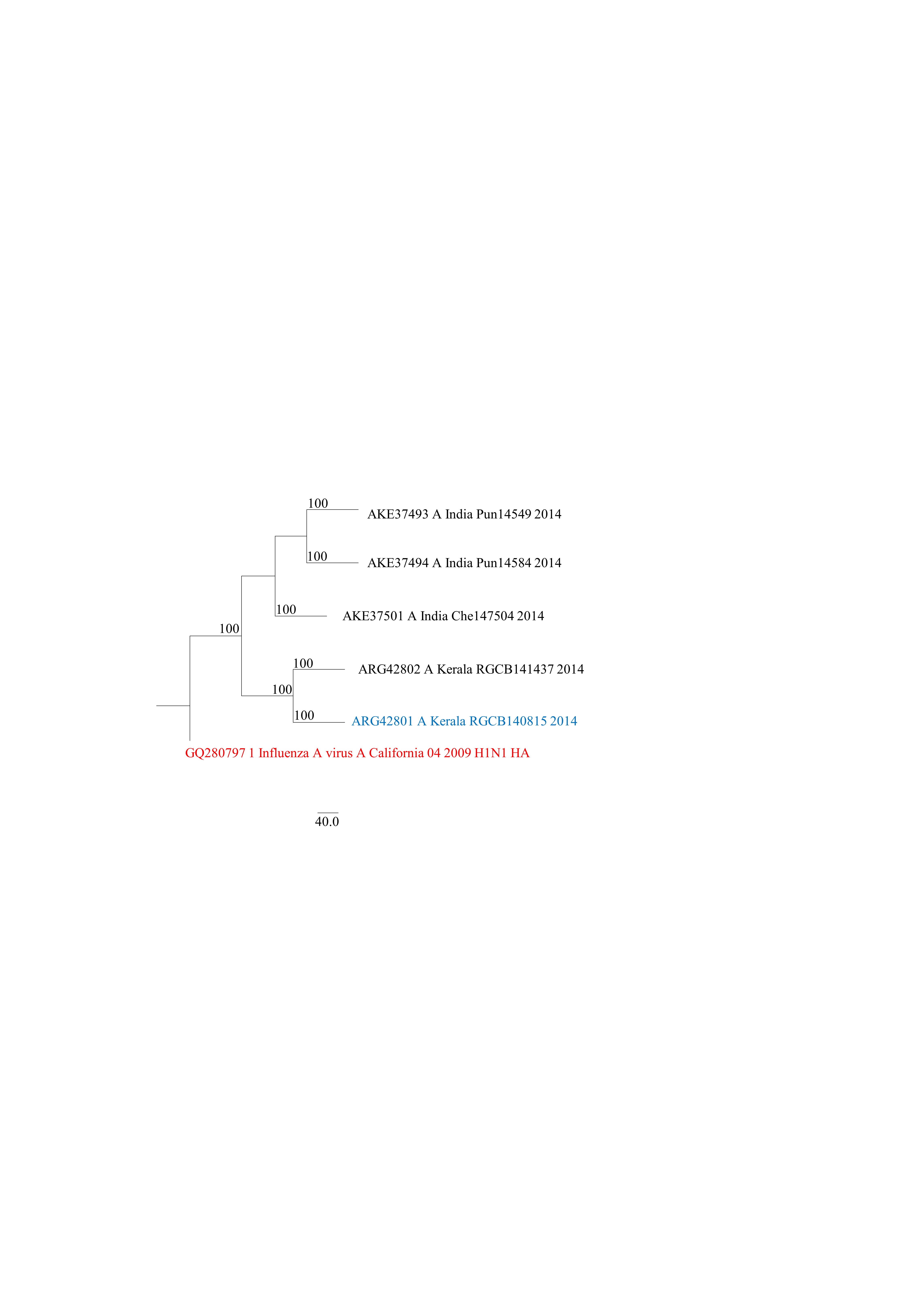


**Supplementary figure 1 (continued):** A phylogenetic tree depicting the relationship between the 2014 Indian H1N1-HA strains and pandemic reference strain A/California/04/2009 is given. Bootstrap values greater than 70% have been given above each node for the maximum parsimony method.


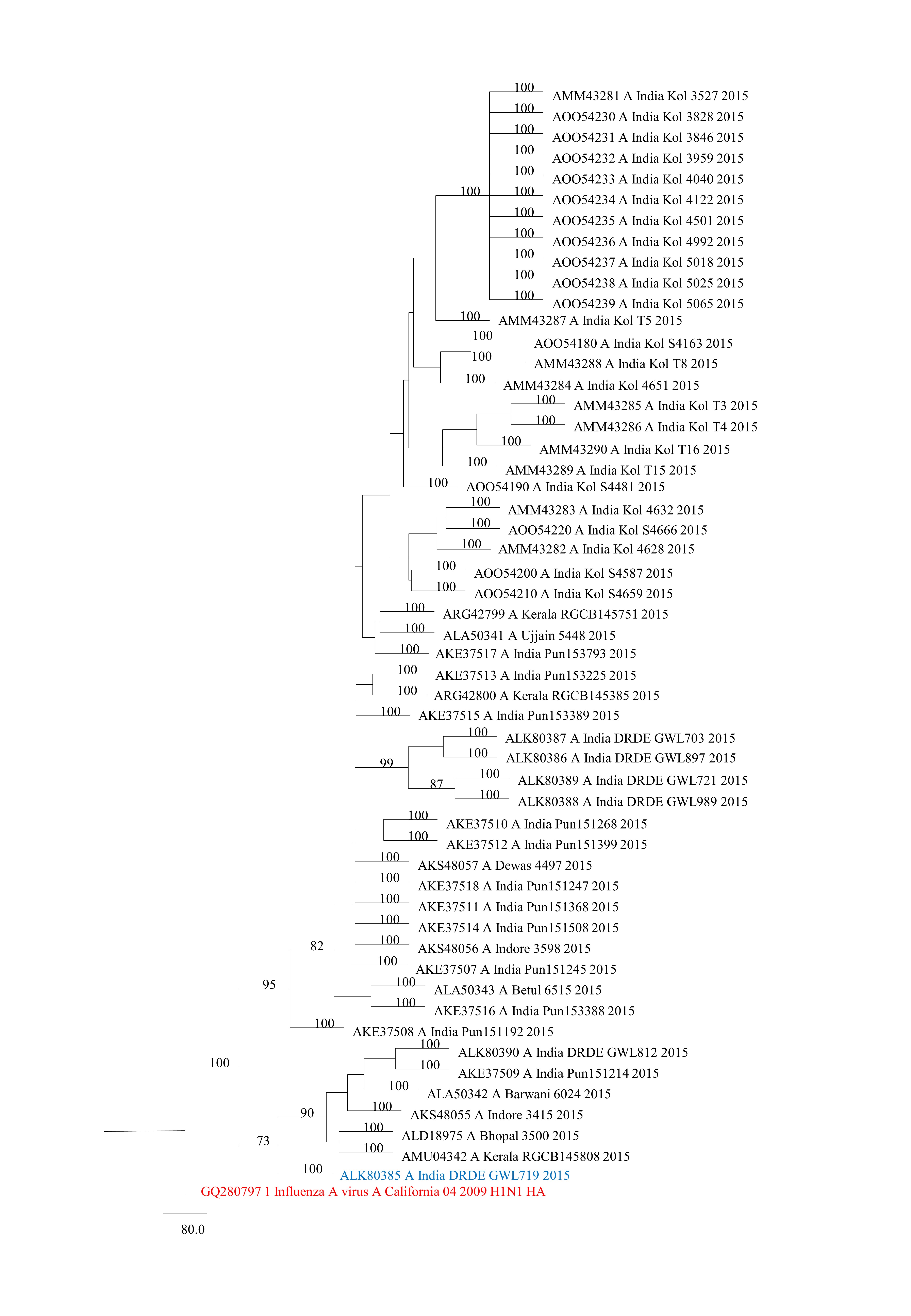


**Supplementary figure 1 (continued):** A phylogenetic tree depicting the relationship between the 2015 Indian H1N1-HA strains and pandemic reference strain A/California/04/2009 is given. Bootstrap values greater than 70% have been given above each node for the maximum parsimony method.


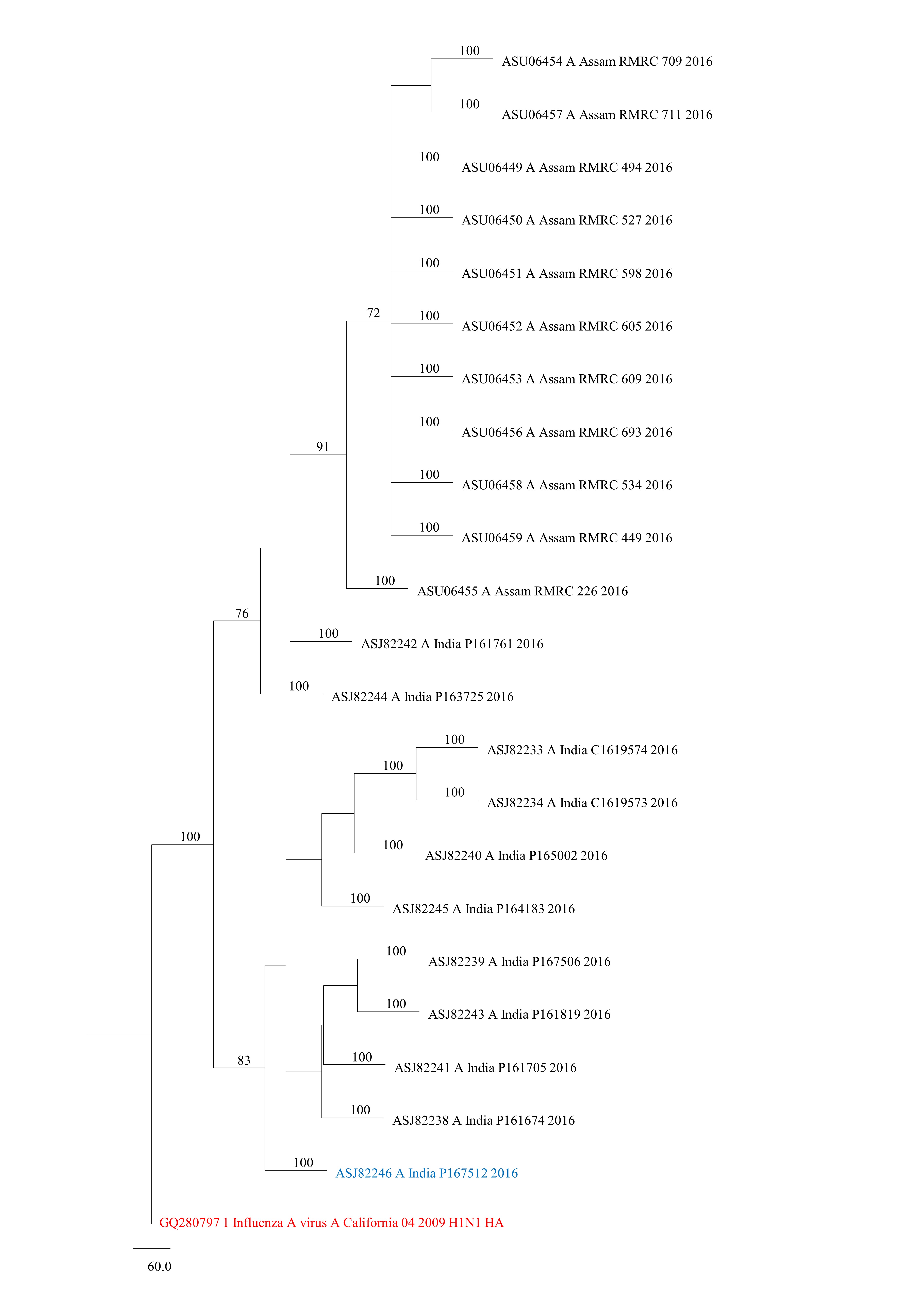


**Supplementary figure 1 (continued):** A phylogenetic tree depicting the relationship between the 2016 Indian H1N1-HA strains and pandemic reference strain A/California/04/2009 is given. Bootstrap values greater than 70% have been given above each node for the maximum parsimony method.


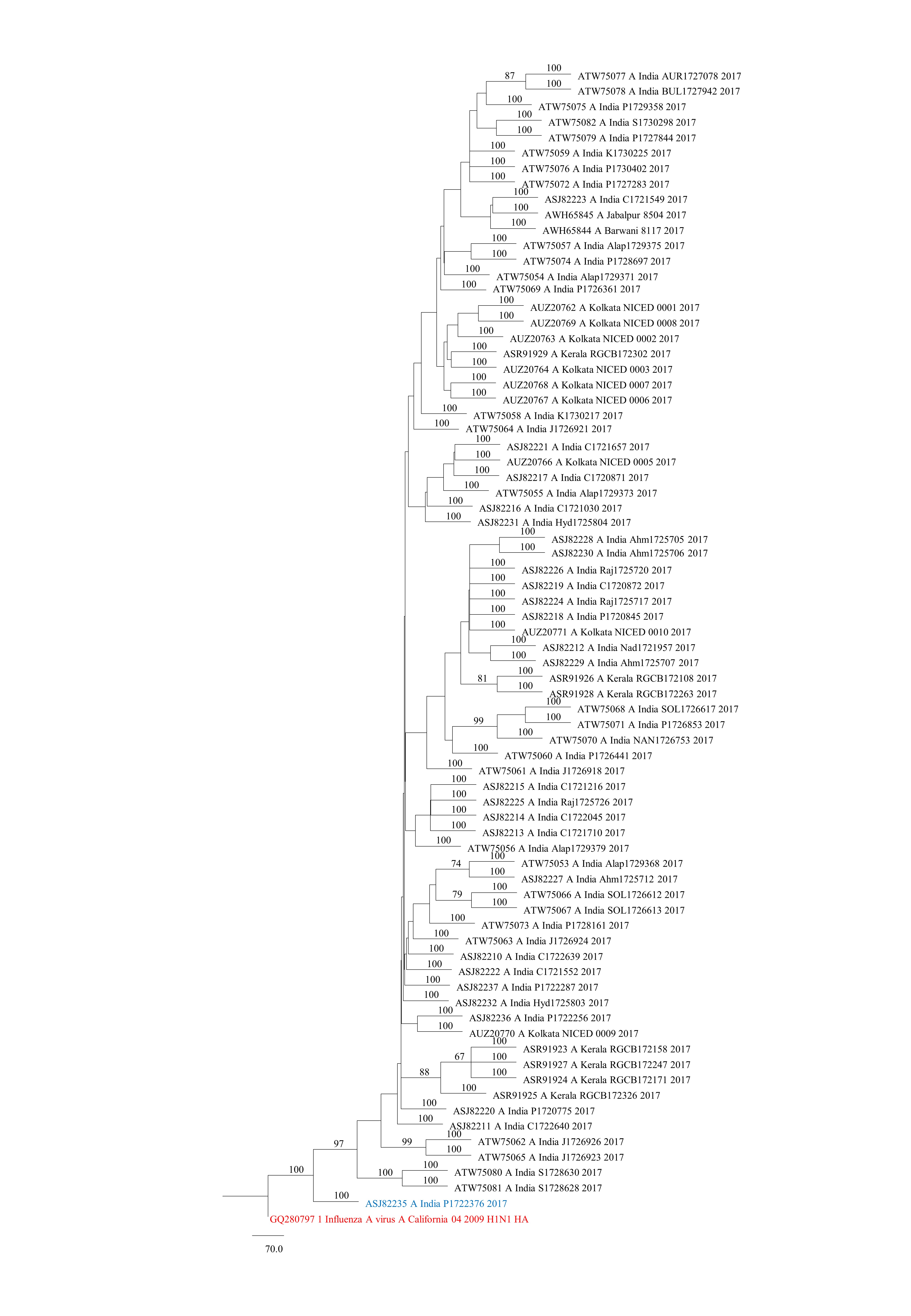


**Supplementary figure 1 (continued):** A phylogenetic tree depicting the relationship between the 2017 Indian H1N1-HA strains and pandemic reference strain A/California/04/2009 is given. Bootstrap values greater than 70% have been given above each node for the maximum parsimony method.


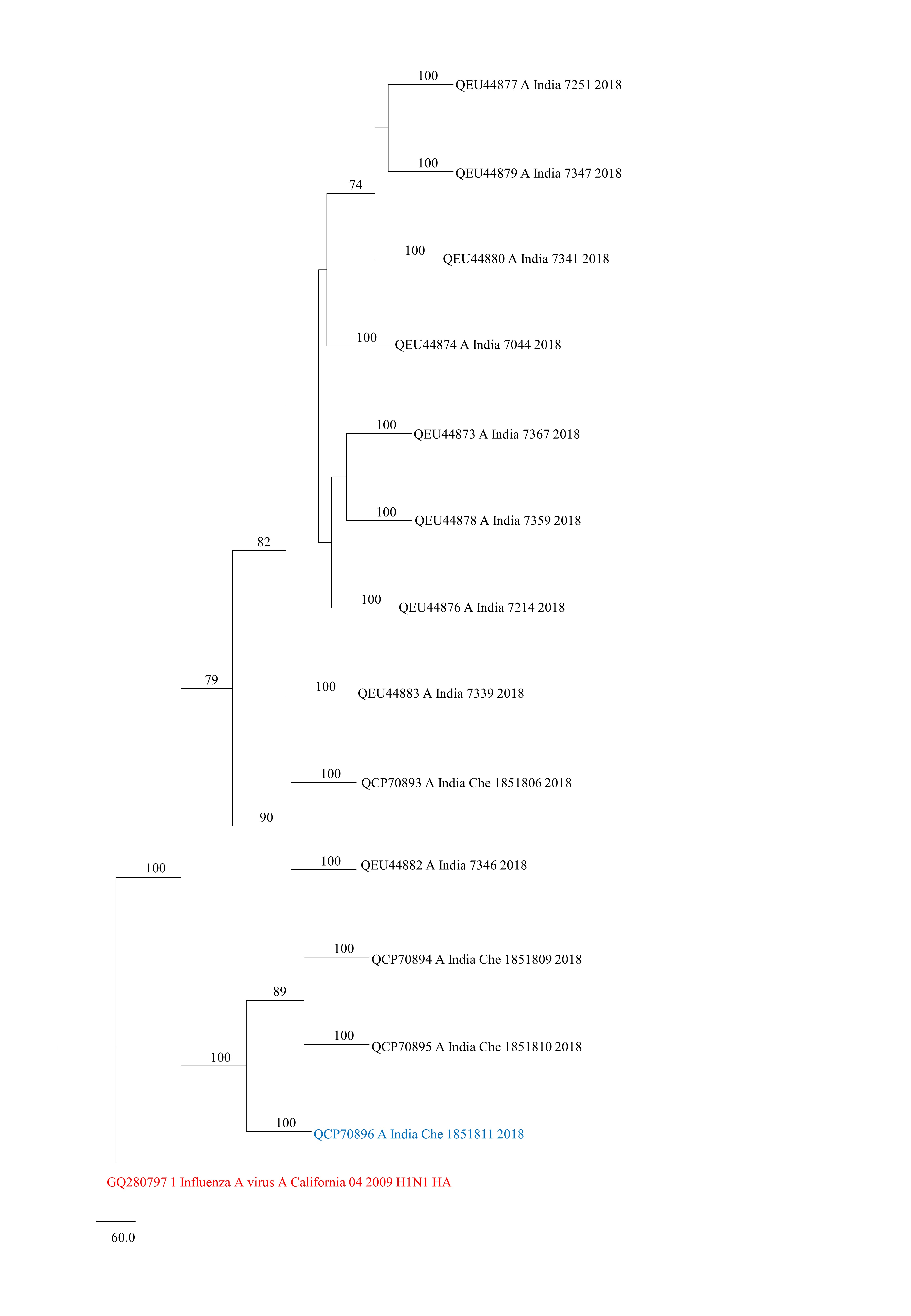


**Supplementary figure 1 (continued):** A phylogenetic tree depicting the relationship between the 2018 Indian H1N1-HA strains and pandemic reference strain A/California/04/2009 is given. Bootstrap values greater than 70% have been given above each node for the maximum parsimony method.
